# Suppression of Inflammatory Demyelinaton and Axon Degeneration through Inhibiting Kv3 Channels

**DOI:** 10.3389/fnmol.2017.00344

**Published:** 2017-10-26

**Authors:** Peter Jukkola, Yuanzheng Gu, Amy E. Lovett-Racke, Chen Gu

**Affiliations:** ^1^Biomedical Sciences Graduate Program, Wexner Medical Center, The Ohio State University, Columbus, OH, United States; ^2^Department of Biological Chemistry and Pharmacology, Wexner Medical Center, The Ohio State University, Columbus, OH, United States; ^3^Department of Microbial Infection and Immunity, Wexner Medical Center, The Ohio State University, Columbus, OH, United States

**Keywords:** voltage-gated K^+^ channel, multiple sclerosis, 4-aminopyridine, experimental autoimmune encephalomyelitis, radial astroglia, brain derived neurotrophic factor

## Abstract

The development of neuroprotective and repair strategies for treating progressive multiple sclerosis (MS) requires new insights into axonal injury. 4-aminopyridine (4-AP), a blocker of voltage-gated K^+^ (Kv) channels, is used in symptomatic treatment of progressive MS, but the underlying mechanism remains unclear. Here we report that deleting Kv3.1—the channel with the highest 4-AP sensitivity—reduces clinical signs in experimental autoimmune encephalomyelitis (EAE), a mouse model for MS. In Kv3.1 knockout (KO) mice, EAE lesions in sensory and motor tracts of spinal cord were markedly reduced, and radial astroglia were activated with increased expression of brain derived neurotrophic factor (BDNF). Kv3.3/Kv3.1 and activated BDNF receptors were upregulated in demyelinating axons in EAE and MS lesions. In spinal cord myelin coculture, BDNF treatment promoted myelination, and neuronal firing via altering channel expression. Therefore, suppressing Kv3.1 alters neural circuit activity, which may enhance BNDF signaling and hence protect axons from inflammatory insults.

## Introduction

Multiple sclerosis (MS) is an inflammatory demyelinating disease of the central nervous system (CNS) with unknown origin, involving both axon demyelination and degeneration (Ferguson et al., 1997; Trapp et al., 1998; Waxman, 2006; Dutta and Trapp, 2011). MS lesions are observed in different brain regions (Lucchinetti et al., 2000; Kutzelnigg et al., 2005; Ng et al., 2013), leading to various symptoms. How these lesions are formed and potentially repaired at different pathogenic stages still remains a mystery. Dalfampridine, a sustained-release form of 4-aminopyridine (4-AP) which is a non-specific blocker of voltage-gated K^+^ (Kv) channels, is used for symptomatic treatment of MS (Hayes, 2004; Judge and Bever, 2006; Goodman et al., 2009; Espejo and Montalban, 2012). The treatment has clear beneficial effects on patients with chronic primary progressive MS, including significant improvement of walking. Long-term usage of dalfampridine appears to be safe and beneficial for some MS patients, but has little effect on MS progression (Cameron et al., 2014; Ruck et al., 2014). Moreover, 4-AP may cause side effects (Judge and Bever, 2006), since Kv channel isoforms sensitive to 4-AP are broadly expressed in the CNS and involved in different neurophysiological functions (Vacher et al., 2008; Gu and Barry, 2011).

Despite intensive research, the target and mechanism of the 4-AP treatment are still not completely understood. This is because there are more than 40 different genes encoding Kv channels and about a dozen of them can be blocked by 4-AP with different efficacies (Judge and Bever, 2006; Gu and Barry, 2011). A number of studies showed that Kv1.3 channels (with 4-AP IC_50_ = 195 μM) are expressed in activated immune cells in mice, and that blocking Kv1.3 activity suppresses the immune response and reduces severity of experimental autoimmune encephalomyelitis (EAE), a mouse model for MS (Chandy et al., 2004; Rus et al., 2005; Gocke et al., 2012). However, the rapid action of 4-AP on MS patients appears to be neurological. Kv1.1 and Kv1.2 (with 4-AP IC_50_ = 590 μM) play a key role in controlling axonal excitability (Judge and Bever, 2006; Gu and Gu, 2011). In myelinated axons, Kv1.1 and Kv1.2 are clustered in juxtaparanodal (JXP) regions under the myelin sheath to prevent repetitive firing of action potentials (Hille, 2001; Debanne, 2004; Gu and Barry, 2011). They also reduce the excitability in premyelinated axons during early development and in demyelinated axons during diseases (Sinha et al., 2006). However, Kv1.1 and Kv1.2 knockout (KO) mice exhibit frequent epileptic seizures and can barely survive over a month (Smart et al., 1998; Brew et al., 2007). Therefore, blocking Kv1.1 and Kv1.2 likely increases the overall excitability of neural circuits, but may cause severe side effects at the same time, including seizures (Goodman et al., 2009). Importantly, the peak serum concentrations of 4-AP in MS patients and control subjects after high, but safe, doses could reach around 1 μM, a very low concentration (Davis et al., 1990; Stefoski et al., 1991; Hayes et al., 2003). It is still possible that some regions in the brain can have higher local concentrations of 4-AP after the treatment. Nonetheless, 4-AP may only activate its most sensitive targets during the treatment of MS patients. However, the role of Kv3.1—the K^+^ channel with the highest 4-AP sensitivity—in MS or its mouse model EAE, has never been investigated.

Kv3 channels (Kv3.1-Kv3.4) are more sensitive to 4-AP than any other Kv channels. In particular, Kv3.1's 4-AP sensitivity is the highest among 4 Kv3 isoforms, and its IC_50_ (~29 μM) is ~6 to 20-fold lower than those of Kv1.1-Kv1.3 (Judge and Bever, 2006). Among the 4 Kv3 channel subfamily members, Kv3.1 and Kv3.2 carry sustained currents, while Kv3.3 and Kv3.4 carry transient currents (Rudy and McBain, 2001). Alternative splicing exclusively occurs at their C-termini, which regulates channel trafficking but not activity (Rudy and McBain, 2001; Xu et al., 2007). Axonal targeting and unique biophysical properties of Kv3 channels—high activation threshold, fast activation/deactivation kinetics—allow neurons to fire action potentials at high frequencies (Gu et al., 2012, 2013). Importantly, Kv3.1 KO mice are viable, fertile, and largely normal (Ho et al., 1997), and display increased spontaneous mobility (Joho et al., 2006), allowing us to examine the role of Kv3.1 in EAE progression.

Besides the role of Kv1.3 in activated immune cells, how Kv channels may affect lesion formation and repair is completely unknown. On the other hand, axonal injury in MS and EAE lesions appears to involve alterations of intrinsic neuronal activities. Upregulation of several types of ion channels in demyelinated axons has been observed, which directly or indirectly causes excessive increase of intra-axonal Ca^2+^ and eventually axon degeneration. Among such aberrantly regulated ion channels are several isoforms of voltage-gated Ca^2+^ (Cav) and Na^+^ (Nav) channels, acid-sensing ion channel 1 (ASIC1) and the transient receptor potential melastatin 4 (TRPM4) channel (Kornek et al., 2001; Craner et al., 2004; Friese et al., 2007; Vergo et al., 2011; Schattling et al., 2012). Blocking these channels could prevent axon degeneration (Friese et al., 2007; Waxman, 2008; Morsali et al., 2013). However, their pathogenic roles remain unclear. More importantly, given the critical role of excessive intra-axonal Ca^2+^ level in axonal damage, it would be beneficial to identify the endogenous mechanisms that can inhibit and/or even reverse this damaging action, for example, via activating a Kv channel to prevent depolarization and hence to limit Ca^2+^ influx.

Demyelination and axonal degeneration can be regulated by extrinsic factors. Radial glia play a key role in neurogenesis, neuronal migration, and gliogenesis during CNS development (McDermott et al., 2005; Kriegstein and Alvarez-Buylla, 2009). Our recent studies showed that radial glia in the adult CNS, cerebellar Bergmann glia, are activated by both inflammatory insults and imbalance of neural activity (via Kv3.1 deletion) (Jukkola et al., 2013). Despite an unknown role of radial glia in EAE and MS progression, these cells can release various factors during inflammation, such as brain derived neurotrophic factor (BDNF). BDNF is a key neurotrophic factor expressed throughout the CNS, promoting neuronal survival, differentiation and myelination (Huang and Reichardt, 2001). BDNF has been found in different cells in MS and EAE lesions mediating complicated functions including neuroprotection (Makar et al., 2008; Linker et al., 2015). Besides its expression in neurons and glia, BDNF was reported to be expressed by immune cells as well (Kerschensteiner et al., 1999). However, using inducible BDNF KO and adoptive transfer EAE, a recent study showed that CNS (but not immune system) cells were the mediating source of BDNF's protective effects on axons at the early stage of EAE (Lee et al., 2012). Therefore, a better understanding of the roles of radial astroglia and BDNF in EAE/MS may lead to new strategies of neuroprotection and repair for treating progressive MS.

In the present study, we induced chronic EAE, an established model for MS, in Kv3.1 KO mice, and found significantly reduced EAE signs at both the peak and late stages. Anatomical studies revealed reduced EAE lesions in sensory and motor tracts of the spinal cord in Kv3.1 KO mice, compared to their wildtype (WT) littermates. This is not likely to be a result of Kv3.1 KO in activated immune cells, since these cells from Kv3.1 KO mice were as effective as those from WT mice in adoptive transfer EAE. In contrast, our findings suggest that Kv3.1 deletion activates the BDNF signaling pathway in radial astroglia to prevent demyelination and axonal degeneration. Interestingly, physical exercise, which voluntarily increases the activity of motor neural circuits, is known to increase BDNF levels in the CNS (Ying et al., 2005; Ding et al., 2011), and can benefit MS patients, whereas blocking or deleting Kv channels increases the activity of neural circuits involuntarily. Taken together, our results provide new mechanistic insights into the benefit of both voluntary and involuntary nerve activities on lesion reduction and repair in inflammatory demyelinating diseases.

## Materials and methods

### Reagents and antibodies

The nuclear (Hoechst 33342) and lipophilic dyes (Fluoromyelin-green and Fluoromyelin-red) were purchased from Invitrogen (Carlsbad, CA, USA). The following antibodies were used in our study: rabbit polyclonal anti-Kv3.1, anti-Kv3.3 and anti-AQP4 antibodies (Alomone Labs, Jerusalem, Israel); rabbit polyclonal anti-BDNF and anti-pan-Trk antibodies (Santa Cruz Biotechnology, Dallas, TX); rabbit polyclonal anti-P75 antibody (a kind gift from Dr. Sung-Ok Yoon, The Ohio State University, Columbus, OH); rabbit polyclonal anti-TrkB (phospho-Y816) antibody (AbCAM, Cambridge, MA, USA); rat monoclonal anti-MBP antibody (Millipore, Billerica, MA); goat polyclonal anti-GFAP antibody (AbCAM); mouse monoclonal anti-Kv1.2, anti-Kv2.1 and anti-Ankyrin-G antibodies (Neuromab, Davis, CA); chicken polyclonal anti-Vimentin antibody (Millipore); Alexa488-, Alexa 647-, Dylight 488-, Dylight 649-, Cy3-, and Cy5-conjugated secondary antibodies (Jackson ImmunoResearch Laboratories, West Grove, PA, USA). Myelin oligodendrocyte glycoprotein (MOG) peptide 35-55 (MEVGWYRSPFSRVVHLYRNGK) was purchased from Pro-Spec (Rehovot, Israel). Recombinant BDNF peptide (mature form) was purchased from Sigma-Aldrich (St. Louis, MO). Ground inactivated mycobacteria tuberculosis H37Ra and Incomplete Freund's Adjuvant were from Difco Laboratories (Detroit, MI, USA).

### Mouse lines and induction of chronic EAE

All animal experiments were conducted in accordance with the NIH Animal Use Guidelines and approved (2008A0177-R3) by Institutional Animal Care and Use Committee (IACUC) of the Ohio State University. The Kv3.1 KO mouse line was kindly provided by Dr. R. Joho at UT Southwestern Medical Center (Ho et al., 1997; Sanchez et al., 2000; Hurlock et al., 2009). The Kv3.1 KO mice were backcrossed with C57BL/6 for more than 10 generations, maintained, and genotyped as previously described (Barry et al., 2013). Chronic EAE was induced in 8–12-week-old female Kv3.1 KO (only Kv3.1^−/−^ homozygous KO mice were used throughout this entire study, 9 mice total) mice and WT littermates (8 mice total), according to previously published methods (Jukkola et al., 2012, 2013). Briefly, myelin oligodendrocyte glycoprotein (MOG) peptide 35–55 (1 mg/ml final concentration) was emulsified in sterile-filtered PBS and Complete Freund's Adjuvant (CFA) containing 2 mg/ml (final concentration) ground inactivated mycobacteria tuberculosis H37Ra. Mice were immunized with 100 μl of MOG/CFA or CFA only (control) by subcutaneous injection at four sites in the belly and in each hind footpad. Pertussis toxin was administered by tail-vein injection at 0 and 2 days post-immunization. Disease progression was monitored by daily clinical scoring on a scale of 0–6 [0 = no symptoms, 1 = loss of tail tone, 2 = hindlimb paresis, 3 = moderate paralysis, 4 = paraplegia (complete hindlimb paralysis), 5 = quadriplegia, 6 = death or moribund state]. Grade 6 animals were removed from the study.

### Adoptive transfer EAE and ELISA

Kv3.1 KO mice (*n* = 3) and WT littermates (*n* = 3) were immunized with MOG/CFA as described above to develop chronic EAE. At 14 DPI when clear EAE signs were observed, their spleens and lymph nodes were harvested and lymphocytes were isolated. The lymphocytes were cultured and stimulated with MOG_35−55_ (2 μg/ml) for 3 days *in vitro*. The levels of IFNγ, IL-17, IL-10, and GM-CSF were measured from the supernatants of these cultured cells from WT and Kv3.1 KO mice, using ELISA (Huss et al., 2013; Lee et al., 2013). Then, 10 × 10^6^ cells were injected i.p. into WT C57BL/6 mice to develop adoptive transfer EAE. Cells from Kv3.1 KO mice and WT littermates were injected into 6 and 7 WT mice, respectively. EAE signs were monitored as described above for chronic EAE.

### Cardiac perfusion, tissue fixation, and sectioning

Mouse tissues were collected and processed according to previously published method with minor modification (Jukkola et al., 2012). In brief, mice were deeply anesthetized with avertin and perfused through the heart with 20–30 ml ice-cold PBS followed by 20 ml 4% formaldehyde in PBS (FA/PBS). The brain and spinal cord of EAE mice were carefully removed and post-fixed for 1 h in 4% FA/PBS. Tissues were cryoprotected in 30% sucrose for at least 24 h, cut into 3-mm blocks using an acrylic brain matrix (Braintree Scientific, Braintree, MA, USA), embedded in optimal cutting temperature (OCT) media (Sakura Finetek USA, Inc., Torrance, CA, USA), and stored at −80°C until sectioning. The tissue blocks were cut with a Microm HM550 cryostat (Thermo Scientific, Waltham, MA, USA) and the 40-μm sections were collected on Superfrost Plus microscope slides (FisherScientific, Pittsburgh, PA, USA) for storage at −20°C.

### Immunofluorescent staining

Sections of mouse brain and spinal cord were stained according to previously published methods (Jukkola et al., 2012). In brief, sections were permeabilized in PBS/1% Triton X-100 for 1 h at room temperature, blocked with 2.5% normal donkey or goat serum (matched with secondary antibody host) for 1 h, and incubated with primary antibodies in the blocking buffer overnight at 4°C. The next day, the sections were rinsed 10 × 5 min, incubated with proper secondary antibodies in the blocking buffer for 2–3 h, counterstained in Hoechst 33342 and/or Fluoromyelin for 5 min, and again rinsed 10 × 5 min (all at room temperature). Slides were coverslipped with tris-buffered Fluoro-Gel mounting media (Electron Microscopy Sciences, Hatfield, PA, USA). Staining with each primary antibody was performed at least twice from 3 to 6 mice per experimental cohort.

### Fluorescence light microscope and image capture

Procedures of fluorescence microscopy and image analysis were described in our published papers (Gu et al., 2006, 2012; Jukkola et al., 2012). Images were captured with a Spot CCD camera RT slider (Diagnostic Instruments, Sterling Heights, MI, USA) in a Zeiss Axiophot upright microscope using 10×/0.30 and 20×/0.50 Plan Apo objectives and saved as 16-bit TIFF files. Exposure times were controlled so that the pixel intensities in the tissues of interest were below saturation, and the same exposure time was used for each group within one experiment. Raw images were used for measurements and quantifications. For representative images in figures, image brightness and contrast were adjusted using Adobe Photoshop 7.0 (Adobe Systems Incorporated, San Jose, CA, USA).

### Image quantification

Images were analyzed with MetaMorph (Molecular Devices, Downingtown, PA, USA) and Sigmaplot 12.0 (Systat Software, Inc., Chicago, IL, USA) for fluorescence intensity quantification and statistical testing. Staining intensities for BDNF, GFAP, and Vimentin were obtained with region measurement in MetaMorph by sampling and automatically calculating the average pixel intensities of small circles (~50-pixel area) drawn on astrocyte soma and processes (10–20 sampled per image), and the values were averaged for each image. Kv3.1, Kv3.3, and TrkB staining intensities were similarly determined along axons with the line measurement, and were averaged over the axons traced. The background intensity of each section was measured and subtracted from the astrocyte or axonal staining intensity for each image. To account for variations in staining intensity from images obtained in different staining experiments, the data were normalized to controls stained side-by-side. The values of normalized fluorescence intensity in images were expressed as mean ± SEM for each experimental group. Statistical significance was determined using an unpaired Student's *t*-test for comparing two groups, or using One-Way ANOVA followed by Dunnett's test for comparing two or more experimental groups to one control group.

### Confocal microscopy and 3D reconstruction

The procedure was described in our recent papers (Jukkola et al., 2012, 2013). High-magnification confocal images were captured with a Leica TCS SL confocal imaging system (Leica Microsystems, Mannheim, Germany), using a 100× HCX Plan Apo CS oil immersion objective (numerical aperture = 1.40). Each image was averaged over four scans in linescan mode. Images were saved as 8-bit TIFF files and adjusted for brightness and contrast using Adobe Photoshop 7.0. For 3D reconstruction, z-stacks of images were collected with a 0.5-μm slice interval. Collapsed images were generated using a maximum intensity projection of the z-stack. For each 3D image, the z-stack was visualized in three-dimensional cross sections, and the cross-bars were centered on pertinent features of the image. The three one-dimensional images were then exported together as an 8-bit TIFF file. Supplementary movies were created using the Maximum Projection with Animation tool, and were adjusted for brightness and contrast within the Leica Confocal Software (v2.61). A maximum intensity projection of the z-stack was rotated 90 degrees around the Y-axis in a series of 18 steps and exported as an AVI file.

### Western blotting

Mice were euthanized with carbon dioxide. Brain and spinal cord tissues were quickly removed, flash-frozen in liquid nitrogen, and stored at −80°C until use. The tissue samples were weighed and homogenized in the lysis buffer containing 50 mM Tris-Cl (pH 7.4), 150 mM NaCl, 1% Triton X-100, 1 mM EDTA, and protease inhibitors including aprotinin, leupeptin, pepstatin A, and phenylmethanesulfonyl fluoride (PMSF), being rocked for 2 h at 4°C. The samples were centrifuged for 30 min at 14,000 rpm at 4°C to collect the supernatant fraction for solubilized proteins. Protein samples were mixed with an equal volume of 2× sample buffer containing 5% β-mercaptoethanol (Sigma), resolved by SDS-PAGE, and transferred to a PVDF membrane (GE Healthcare, Piscataway, N, USA) for western blotting, as previously described (Jukkola et al., 2012). The membrane was rinsed in TBST, incubated in 5% dry milk/TBST blocking solution for 1 h at room temperature, and then incubated with the primary antibody in the blocking solution overnight at 4°C. Membranes were then washed in TBST 4 × 10 min, incubated with a horseradish peroxidase-conjugated secondary antibody in the blocking solution for 1 h, and washed again in TBST 5 × 10 min. Membranes were then incubated in ECL (GE Healthcare) chemiluminescent solution, wrapped in plastic, and exposed to X-ray film (Kodak, Rochester, NY, USA). Developed films were digitized with an Epson 3590 scanner (Epson America, Inc., Long Beach, CA, USA). The total intensities of protein bands were measured and quantified with NIH ImageJ. Background was subtracted for each band. Three mice were included in each condition. All Western blotting experiments were performed three times.

### Myelin coculture of SC neurons

Before myelin coculture, an astrocyte culture was established. The culture media was similar to the plating media that we used for hippocampal neuron culture, consisting of 1× modified essential media (MEM) with Earle's salts (Gibco/Life Technologies) containing 10% fetal bovine serum (Gibco/Life Technologies), Penicillin/Streptomycin (100 units/ml Pen; 100 μg/ml Strep; Gibco/Life Technologies), 1× L-Glutamine (2 mM; Gibco/Life Technologies), and 1× sodium pyruvate (1 mM; Gibco/Life Technologies). Coverslips were precleaned and coated with poly-D-lysine and rat tail collagen according to the previously published method (Gardner et al., 2012). SC from rat embryos (at day 18) (E18; ~2 embryos per one 24-well plate) were removed and cleaned in ice-cold slice dissociation solution (Gardner et al., 2012). SC segments were then incubated in proteinase solution for 15–20 min at 37°C, rinsed twice with 10 ml prewarmed culture media, and dissociated by trituration with a 9-inch pasteur pipette in ~4 ml media. The cell suspension was diluted in culture media and ~0.4 ml was added to each well of 24-well plates containing coverslips. After attachment of cells to the coverslips (~4–6 h), the media was aspirated from each well and replaced with 1 ml fresh plating media. Cultured cells were fed by replacement of 0.5 ml plating media with fresh media every 4 days. Under this culture condition, the majority of surviving cells were astrocytes.

After 2 weeks of culture, astrocytes became a confluent layer on the coverslips. We disassociated cells from SC of second group of rat E18 embryos and suspended them in the plating media. After removing half of the media from the astrocyte culture, we plated suspended cells from E18 SC on top of the astrocyte layer, which was essential for motor neurons to survive and grow. After about 4–6 h, half of the plating media was replaced with myelin coculture media, prepared as previously published (Gardner et al., 2012). The myelin coculture media was then used for all subsequent media changes to replace one-third of the culture media every 3 days.

### BDNF treatment of the myelin coculture

Myelin coculture of spinal cord neurons around 4–5 weeks was treated with mature BDNF for 1 week, with the initial concentration of 100 ng/ml supplemented daily with 20 ng/ml. The initial concentration (100 ng/ml BDNF) was chosen based on published literature describing BDNF treatment in spinal cord motor neuron culture (Loeb and Fischbach, 1997) or dorsal root ganglion culture (Chan et al., 2004). Treated neurons were used for immunostaining and patch clamp recording.

### Voltage- and current-clamp recording from cultured spinal cord neurons

Inward (downward) and outward (upward) currents were determined with voltage-clamp recording on cultured motor neurons as previously described (Gu et al., 2012). Current-clamp recording was performed on the same neurons to examine action potential firing patterns as described (Gu et al., 2012, 2013). Hank's buffer consists of 150 mM NaCl, 4 mM KCl, 1.2 mM MgCl_2_, 10 mg/ml glucose, 1 mM CaCl_2_, 20 mM HEPES (pH 7.4). The internal solution of electrical pipettes contains (in mM) 122 KMeSO_4_, 20 NaCl, 5 Mg-ATP, 0.3 GTP, and 10 HEPES (pH 7.2). Glass pipettes with tip diameter around 1 μm for patch clamp recording were pulled with Model P-1000 Flaming/Brown micropipette puller (Sutter Instrument, Novato, CA, USA). The patch pipette resistance in the bath was about 2–2.5 MΩ. The whole-cell access resistance was between 3 and 5 MΩ. Cells with higher access resistance were discarded. The compensation for series resistance was set >60%. Conductance-voltage relationship (G-V curve) for both inward and outward currents was G = I / (V_m_ − V_rev_). V_rev_ = −95 mV for outward currents and V_rev_ = 70 mV for inward currents, respectively (Rush et al., 2005). The resulting conductance was normalized to the corresponding maximal conductance. Curves were fitted with the following Boltzmann function, G/G_max_ = 1/(1+exp[−(V−V_1/2_)/k]), where G_max_ is the maximal conductance, V_1/2_ is the potential at which the value of the relative conductance is 0.5, and k is the slope factor.

### Immunostaining of post-mortem human brain sections from non-MS controls and MS patients

Post-mortem brain sections (with de-identification) from MS patients and non-MS controls were kindly provided by Dr. David Pitt (Yale University). Human CNS tissue was obtained according to Institutional Review Board-approved protocols. Sections were incubated in xylenes to deparaffinize the tissue, and were rehydrated in 100, 95, 70, and 50% ethanol, followed by ddH_2_O (5 min each solution). Antigen retrieval was performed according to Evers and Uylings (1997) with some modification. Briefly, sections were placed in prewarmed 10 mM Tris-buffered saline (0.9% NaCl), microwaved for 5 min, and allowed to cool in the same solution for 5 min. Fluorescence immunohistochemistry was then performed as normal. To circumvent the problem of notoriously high levels of autofluorescence in human sections, we adopted a Sudan-Black-based quenching method to eliminate autofluorescence (Schnell et al., 1999). After the secondary antibody incubation was complete, the sections were washed 4 times to remove excess antibody. Then the sections were incubated with 2% (w/v) Sudan Black B in 70% Ethanol for 5 min at room temperature. The sections were then washed 5× for 5 min in the normal immunohistochemistry washing buffer. Using this method, we successfully performed double-immunofluorescence staining. Kv channel staining signals in MS lesions and adjacent normal-appearing white matter were compared with non-MS controls. We examined multiple patients with two different types of MS, relapsing-remitting and secondary progressive, and multiple lesions from each patient.

### Statistical analysis

Results were presented as the mean ± SEM. Two-tailed Student's *t*-test was used for comparisons between two groups. One-way ANOVA followed by Dunnett's test was used for comparing two or more groups to one control group. ^*^p < 0.05 and ^**^p < 0.01 were considered statistically significant.

## Results

### Reduction of EAE clinical signs in Kv3.1 KO mice correlates with decreased lesion formation

**Figure 1:**
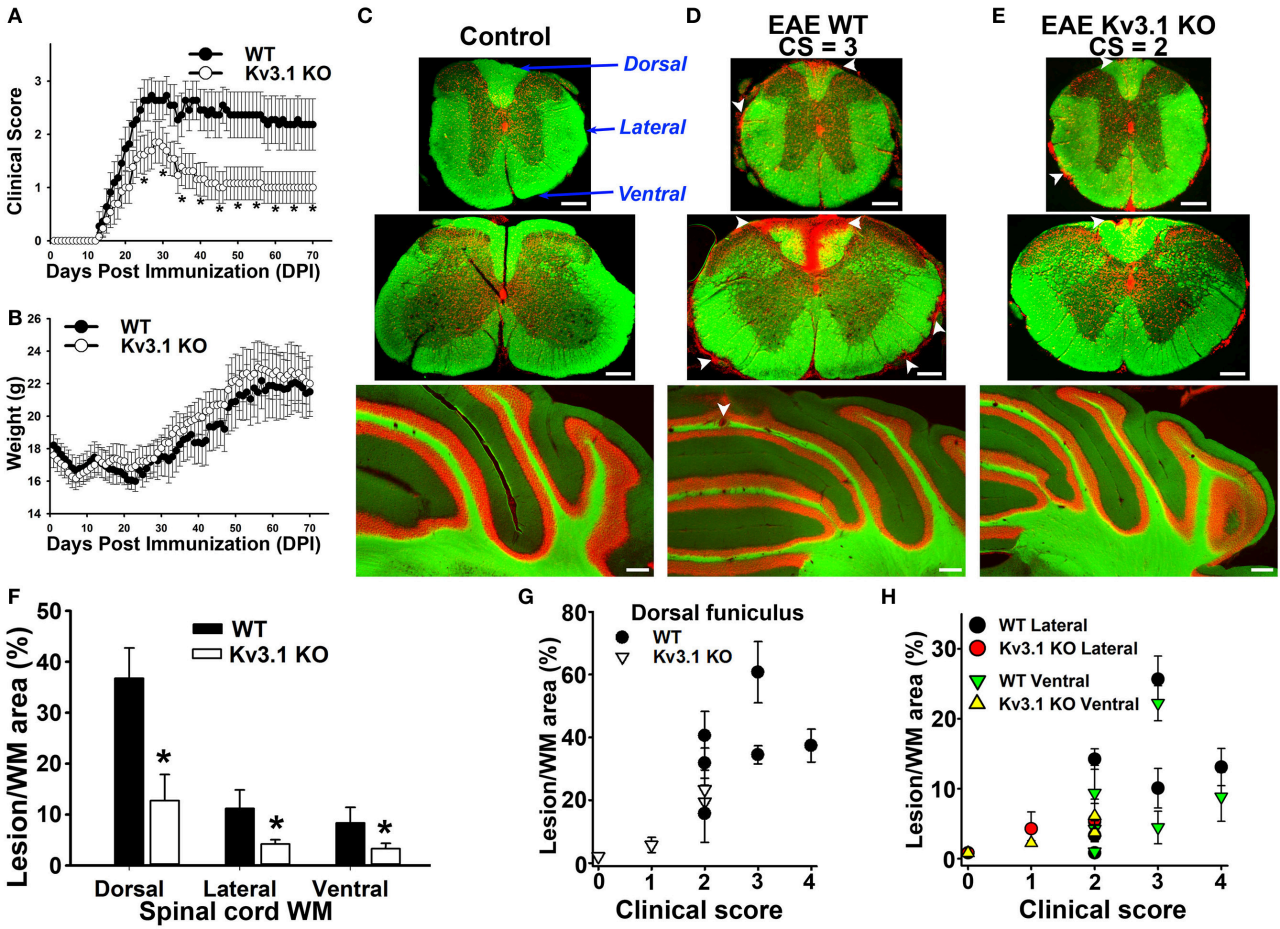
Deleting Kv3.1 channel reduces EAE severity through lesion reduction. **(A)** Reduction of EAE severity in Kv3.1 KO mice (*n* = 9) compared to WT mice (*n* = 8). **(B)** Alterations of mouse body weight during EAE progression. **(C)** EAE lesions in control mice. Cross sections of spinal cord (top, thoracic; middle, cervical) and coronal section of the cerebellum (bottom) were stained with FluoroMyelin Green (FMG; green) and nuclear dye (Hoechst; red). CS, clinical score. Dorsal, lateral, and ventral funiculi were indicated with blue arrows. **(D)** EAE lesions at the late stage in WT mice. **(E)** EAE lesions at the late stage in Kv3.1 KO mice. White arrowheads, lesions. Scale bars, 250 μm. **(F)** The summary of EAE lesion areas in dorsal, ventral and lateral funiculi white matter in WT and Kv3.1 KO mice. EAE mice at 60 DPI were examined. Unpaired *t*–test: ^*^p < 0.05. **(G)** Correlations between clinical scores and lesion areas in WT and Kv3.1 KO mice in dorsal funiculus. **(H)** Correlations between clinical scores and lesion areas in WT and Kv3.1 KO mice in lateral and ventral funiculi. **(C–H)** 6 wild type mice and 4 Kv3.1 KO mice were used. About 20 images for each mouse were used for quantification of lesion areas.

To determine the potential role of Kv3.1 in MS progression including inflammatory demyelination and axon degeneration, we induced monophasic chronic EAE in female Kv3.1 KO mice and WT littermates with myelin oligodendrocyte glycoprotein (MOG) peptide 35–55 as described before (Jukkola et al., 2012, 2013). EAE clinical scores were significantly reduced in Kv3.1 KO mice at both peak and late stages, but not at the onset, compared to their WT littermates (Figure 1A). Body weights of both WT and Kv3.1 KO mice were transiently reduced after immunization and were regained after about 25 days (with a slightly faster rate for Kv3.1 KO mice). However, overall there was no significant difference in weight changes between WT and Kv3.1 KO mice during EAE progression (Figure 1B). This result raised an interesting question: how does deleting Kv3.1 lessen EAE signs? We first considered the following two possible mechanisms. (1) Kv3.1 deletion might reduce EAE severity by altering neural circuits in a compensatory manner, without affecting lesion formation. (2) Kv3.1 deletion might decrease EAE severity by reducing lesion formation through an unknown mechanism.

To determine lesion patterns in the CNS during EAE progression, we systematically examined the spinal cord (SC) and cerebellum of WT and Kv3.1 KO mice using two fluorescent dyes (Figures 1C–E). FluoroMyelin Green (FMG) is a lipophilic dye to label myelin membranes, whereas Hoechst is a nuclear dye to label cell bodies. We used the two dyes to reveal white matter (WM) and gray matter (GM) in CNS tissue sections (Jukkola et al., 2012, 2013). In WT mice at the late EAE stage, clusters of infiltrating cells and loss of myelin staining signals indicated lesion sites in both SC and cerebellar WM (Figure 1D). Compared to their WT littermates, the EAE lesions of Kv3.1 KO mice were significantly fewer in number and smaller in size, at both peak and late stages (Figures 1C–E).

To further quantify the pattern of lesion formation, we examined three different SCWM areas, including dorsal, lateral, and ventral funiculi (Figure 1C). The dorsal funiculus exclusively contains sensory axons, whereas the ventral funiculus contains mostly motor axons and the lateral funiculus contains both sensory and motor axons. In this MOG-induced chronic EAE, we found more lesions located along sensory axonal tracts in the dorsal funiculus, compared to lateral and ventral funiculi (Figures 1D–H). This result is consistent with the fact that both sensory and motor functions are affected in MS patients. In Kv3.1 KO mice, EAE lesions in all three areas were significantly less compared to WT EAE mice, particularly in the ventral funiculus where they were almost completely absent (Figure 1F).

### Immune cells from Kv3.1 KO and WT mice have similar potency in EAE induction

**Figure 2:**
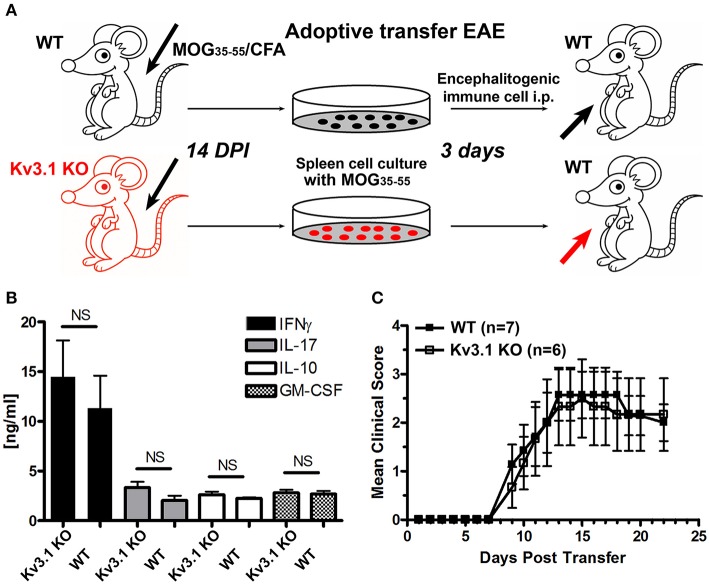
Efficacy of immune cells from Kv3.1 KO mice in adoptive transfer EAE. **(A)** Diagram of adoptive transfer EAE. First, chronic EAE was induced with MOG_35–55_ on both Kv3.1 KO mice and WT littermates. At 14 DPI when clear EAE signs were observed, their splenocytes were removed and cultured *in vitro* with stimulation by MOG_35–55_ for 3 days. These cells were then transferred into WT C57BL/6 mice. **(B)** No significant differences in cytokine production between Kv3.1 KO and WT cells was observed. The levels of IFNγ, IL-17, IL-10, and GM-CSF were measured in the supernatants by ELISA. **(C)** No significant difference in EAE clinical scores was observed between the mice that received WT lymphocytes and those that received Kv3.1 KO lymphocytes.

To determine whether reduced EAE lesions might result from an unknown role of Kv3.1 channel in immune cell activation and proliferation, we performed adoptive transfer EAE to compare the effects of immune cells from Kv3.1 KO mice and their WT littermates (Figure 2A). WT and Kv3.1 KO mice were used to induce chronic EAE with the same procedure as described in Figure 1A. At 14 days post-immunization (DPI) when mice started to exhibit clear EAE signs, their splenocytes were dissected out and cultured *in vitro*. These cells were cultured with further stimulation of MOG_35−55_ peptides for 3 days. ELISA results showed no difference between these immune cells of WT and Kv3.1 KO mice in terms of secreted cytokines, such as interferon gamma (IFNγ), interleukin 17 (IL-17), interleukin (IL-10), and granulocyte-macrophage colony stimulating factor (GM-CSF) (Figure 2B). Then, these cells from either WT or Kv3.1 KO mice were injected into normal C57Bl/6 female mice to induce EAE (Figure 2A). Importantly, after being transferred into mice, these cells were equally potent in EAE induction (Figure 2C). Therefore, reduced EAE lesion formation in Kv3.1 KO mice is unlikely to be mediated by defective immune cells due to the lack of Kv3.1 channels. This is very different from what happens with the Kv1.3 KO mice in EAE (Gocke et al., 2012), where Kv1.3 plays an important role in immune cell proliferation.

### Upregulation of axonal Kv3.1 and Kv3.3 channels in EAE lesion sites

**Figure 3:**
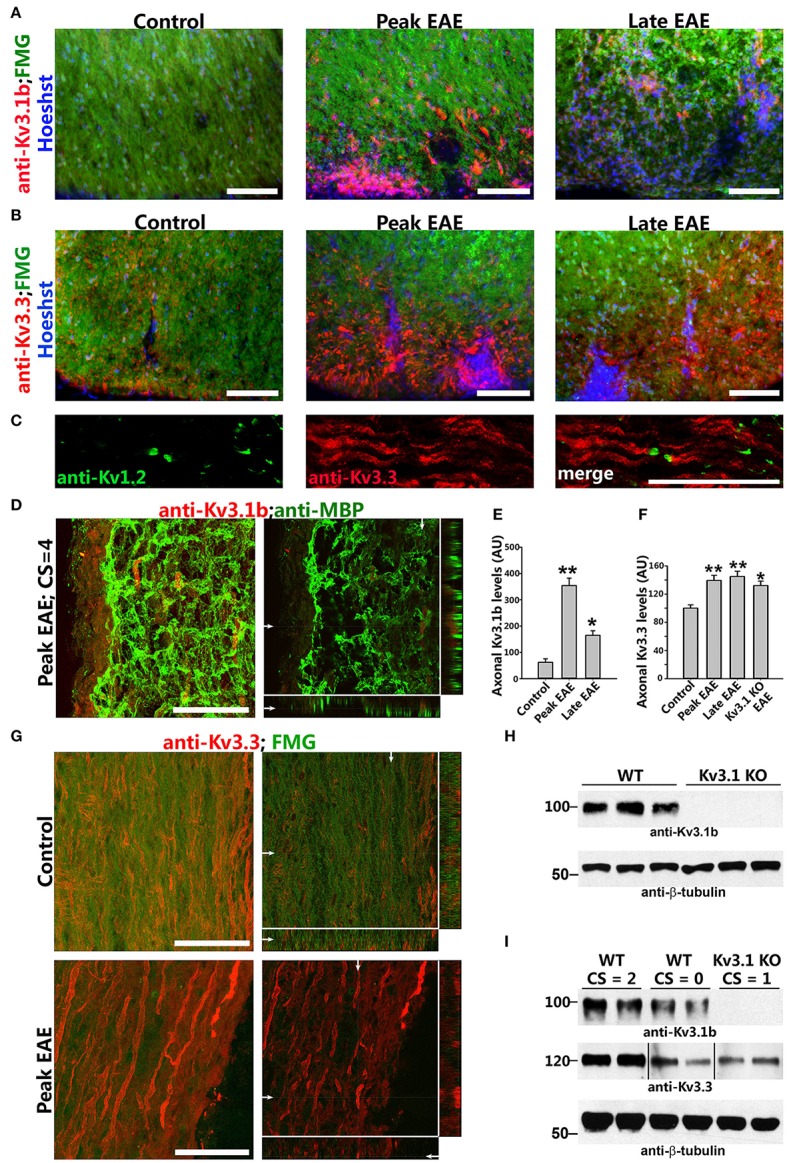
Axonal Kv3.1 and Kv3.3 channels increased during EAE progression. **(A)**, Cross sections of SC ventral funiculus in WT mice at the control, peak, and late EAE stages stained with FMG (green), Hoechst (blue), and an anti-Kv3.1b antibody (red). **(B)** Similar cross sections stained for Kv3.3 (red). **(C)** SC longitudinal section from a control mouse was stained for Kv1.2 (green) and Kv3.3 (red). **(D)** Upregulated Kv3.1 in axonal segments that were partially demyelinated (anti-Kv3.1b in red and anti-MBP in green) at the peak stage of EAE. The high magnification confocal image stack was obtained from a lesion area in a longitudinal SC section. The collapsed 2D image is on the left, and three cross sections are on the right. White arrows, positions for the other two cross sections. **(E)** Summary of axonal levels of Kv3.1b at three different stages of EAE. One-way ANOVA followed by Dunnett's test. ^**^p < 0.01; ^*^p < 0.05. *n* = 50. **(F)** Axonal Kv3.3 levels were upregulated during EAE (anti-Kv3.3 in red and FMG in green). Top, control; bottom, peak EAE. High magnification confocal image stacks were obtained from longitudinal SC sections. The collapsed 2D images are on the left, and 3 cross sections are on the right. Scale bars, 50 μm. **(G)** Summary of axonal levels of Kv3.3 at three different stages of EAE in WT and Kv3.1 KO mice. One-way ANOVA followed by Dunnett's test. ^**^p < 0.01; ^*^p < 0.05. *n* = 50. **(H)** Confirmation of no Kv3.1 expression in Kv3.1 KO mice by Western blotting. **(I)** Alterations of Kv3.1b (middle) and Kv3.3 (bottom) levels during EAE in WT and Kv3.1 KO mice.

To understand how deleting Kv3.1 affects EAE lesion formation, we examined alterations of Kv3 channel expression and targeting in CNS lesion areas in WT EAE mice. Kv3.1 expression was low in SCWM under normal conditions, but increased at both the peak and late stages of chronic EAE. In particular, Kv3.1 level was significantly upregulated in some axonal segments surrounding and within lesion areas (Figures 3A,D,E). Because of potential effects of heterotetramerization and compensation on Kv3.1, we examined alterations of the other three Kv3 (Kv3.2–Kv3.4) channels in SCWM during EAE. Among these three, Kv3.3 was the most highly expressed one in SC and partially colocalized with Kv3.1. Compared to Kv3.1, Kv3.3 channels are more broadly expressed in the CNS (Chang et al., 2007). Studies indicate that while Kv3.3-mediated fast repolarization of Purkinje neurons is critical for normal motor speed, Kv3.1-dependent fast repolarization in deep cerebellar nuclei neurons is crucial for timing or gait patterning (Hurlock et al., 2008, 2009; Zagha et al., 2008). Interestingly, Kv3.3 was expressed in the axons of SCWM under the control condition, and its axonal level markedly increased during EAE at both the peak and late stages (Figures 3B,C,F,G and Movies S1, S2). This is the first report that axonal levels of Kv channels significantly increase in EAE lesion. These results were confirmed by Western blotting reflecting global changes (Figures 3H,I). In naive Kv3.1 KO mice, the total expression level of Kv3.3 in the CNS did not appear to change significantly. Alteration of Kv3.1 and/or Kv3.3 levels changes intrinsic excitability and/or synaptic transmission in some CNS neurons, as reflected by increased motility observed in their single and double KO mice (Joho et al., 2006).

### Upregulation of astrocytic intermediate filaments in Kv3.1 KO mice

**Figure 4:**
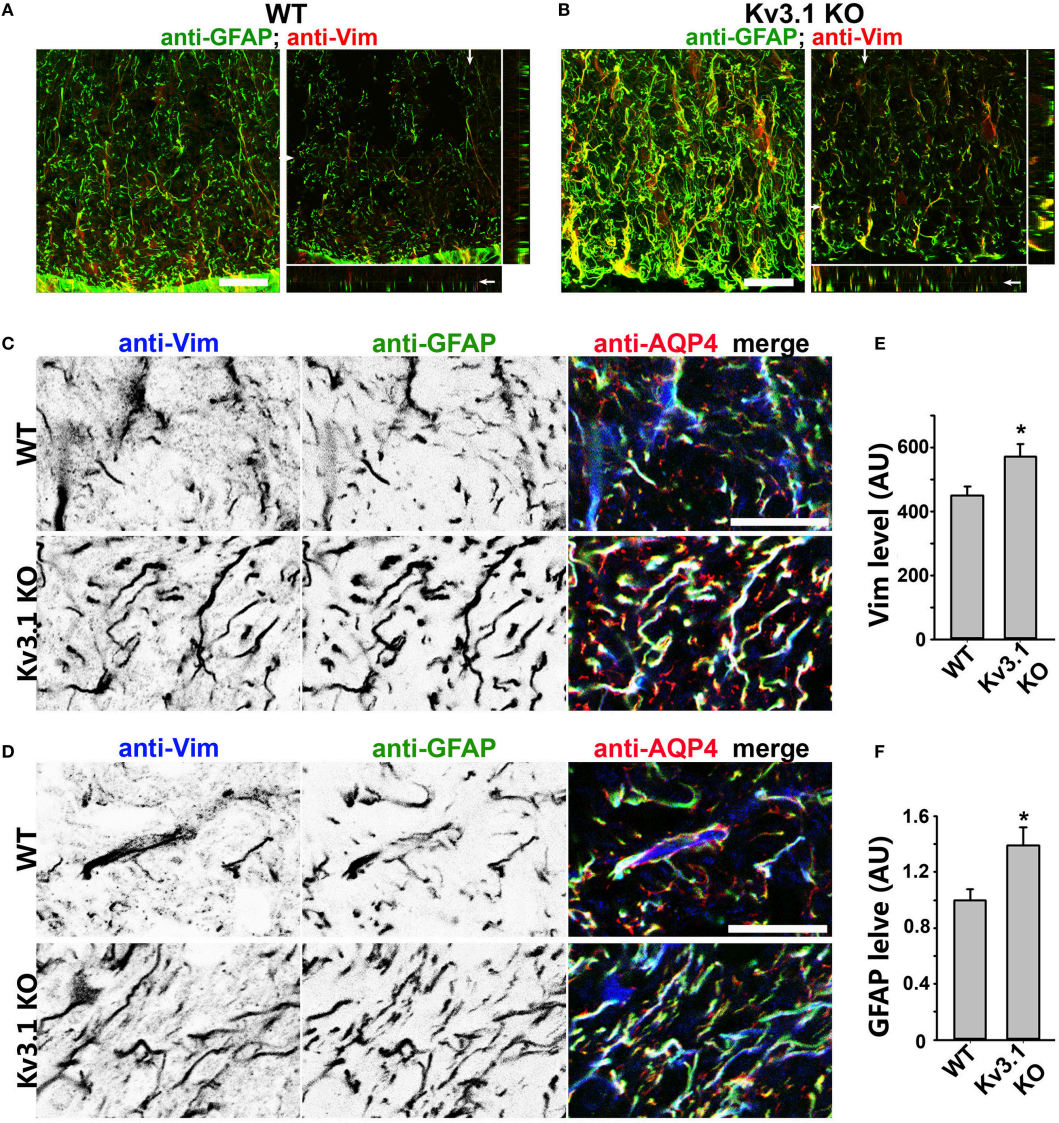
Upregulation of astrocytic intermediate filaments in Kv3.1 KO mice. **(A)** Cross sections of ventral SCWM in WT mice were costained for GFAP (green) and Vim (red). The collapsed 2D image is on the left, and three cross sections are on the right. **(B)** Cross sections of ventral SCWM in Kv3.1 KO mice were costained for GFAP (green) and Vim (red). Scale bars, 200 μm. **(C)**, Increased expression of Vim (inverted gray scale image on the left; blue in the merged image on the right), GFAP (inverted gray scale image in the middle; green in the merged image on the right), and AQP4 (red) in dorsal SCWM of Kv3.1 KO mice (bottom) compared to that of WT mice (top). **(D)** Increased expression of astrocytic proteins as in **(C)** in ventral SCWM. Scale bars, 100 μm. Summary of expression levels of Vim **(E)** and GFAP **(F)** in WT and Kv3.1 KO mice. Unpaired Student *t*–test: ^*^p < 0.05. *n* = 50. The results were from 3 Kv3.1 KO and 4 WT mice.

To determine potential alterations of astrocytes in this process, we examined astrocytic markers in the SC from Kv3.1 KO and EAE mice. This is because astrocytes are known to play critical roles in the pathogenic process of EAE and MS, and our recent studies showed that astrocytes in the adult CNS, including cerebellar Bergmann glia, are activated by both inflammatory insults and imbalance of neural activity (via Kv3.1 deletion) (Jukkola et al., 2013). Here we found that two astrocyte markers, glial fibrillary acidic protein (GFAP) and vimentin (Vim), were markedly upregulated in the SC of Kv3.1 KO mice, although there was no sign of astrocyte proliferation and hypertrophy (Figure 4 and Movies S3, S4). It was reported that GFAP deletion increases EAE severity (Liedtke et al., 1998) and that intermediate filaments including GFAP and Vim are critical for astrocytes' resistance to mechanical stress (Pekny and Pekna, 2004). Thus, the increased GFAP and Vim in Kv3.1 KO mice may increase the rigidness of astrocytes and inhibit the infiltration of immune cells into the CNS.

### Alterations of astrocytic BDNF and its receptors in axons in Kv3.1 KO and EAE mice

**Figure 5:**
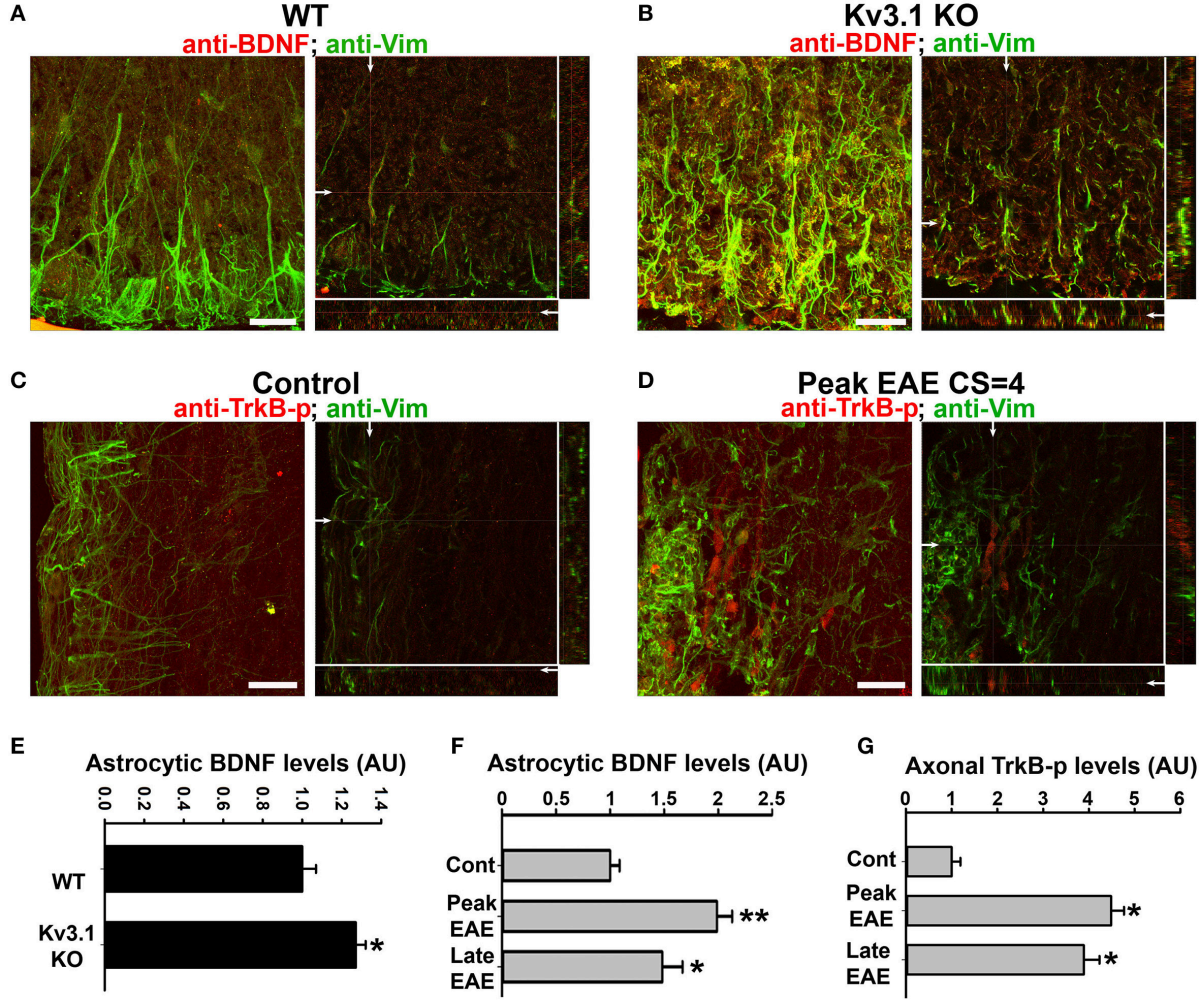
Alterations of BDNF and Trk receptor levels in Kv3.1 KO mice and EAE mice. The levels of BDNF (red) and Vim (green) in SCWM of WT **(A)** and Kv3.1 KO **(B)** mice. High magnification confocal image stacks were obtained from cross sections of ventral SCWM. The collapsed 2D images are on the left, and three cross sections are on the right. Scale bars, 200 μm. The levels of phospho-TrkB (TrkB-p) receptors (red) and Vim (green) in the control **(C)** and peak EAE **(D)** stages of WT mice. The image stacks were captured from longitudinal sections of SCWM. **(E)** The BDNF level significantly increased in Vim-positive astrocytes in SCWM of Kv3.1 KO mice. Unpaired Student *t*–test: ^*^p < 0.05. **(F)** BDNF levels in Vim-positive astrocytes at different stages of EAE in WT mice. **(G)** Axonal pan-Trk levels increased during EAE. One-way ANOVA followed by Dunnett's test in **(F,G)**. ^**^p < 0.01; ^*^p < 0.05. *n* = 50.

To determine whether chemical signaling, in addition to physical constraints, plays an important role in lesion prevention and repair, we examined potential alteration of neurotrophic factors by using both immunostaining and Western blotting. It is known that BDNF can promote axonal outgrowth and neuronal survival in the CNS (Huang and Reichardt, 2001). In particular, it was reported that astrocytic BDNF promoted remyelination through its receptor in a toxin-induced demyelination (cuprizone) model (Fulmer et al., 2014). Interestingly, our studies show that BDNF expression levels significantly increased in SCWM of Kv3.1 KO mice, particularly in the Vim-positive radial astrocytes (Figures 5A,B and Movies S5, S6). In contrast, we did not observe a clear difference in the expression level of another neurotrophic factor, neural growth factor, in the KO mice. Thus, BDNF upregulation was not likely a general effect of all neurotrophic factors in this process. The increased BDNF expression in SC radial astroglia may promote myelination/remyelination and axonal regeneration.

**Figure 6:**
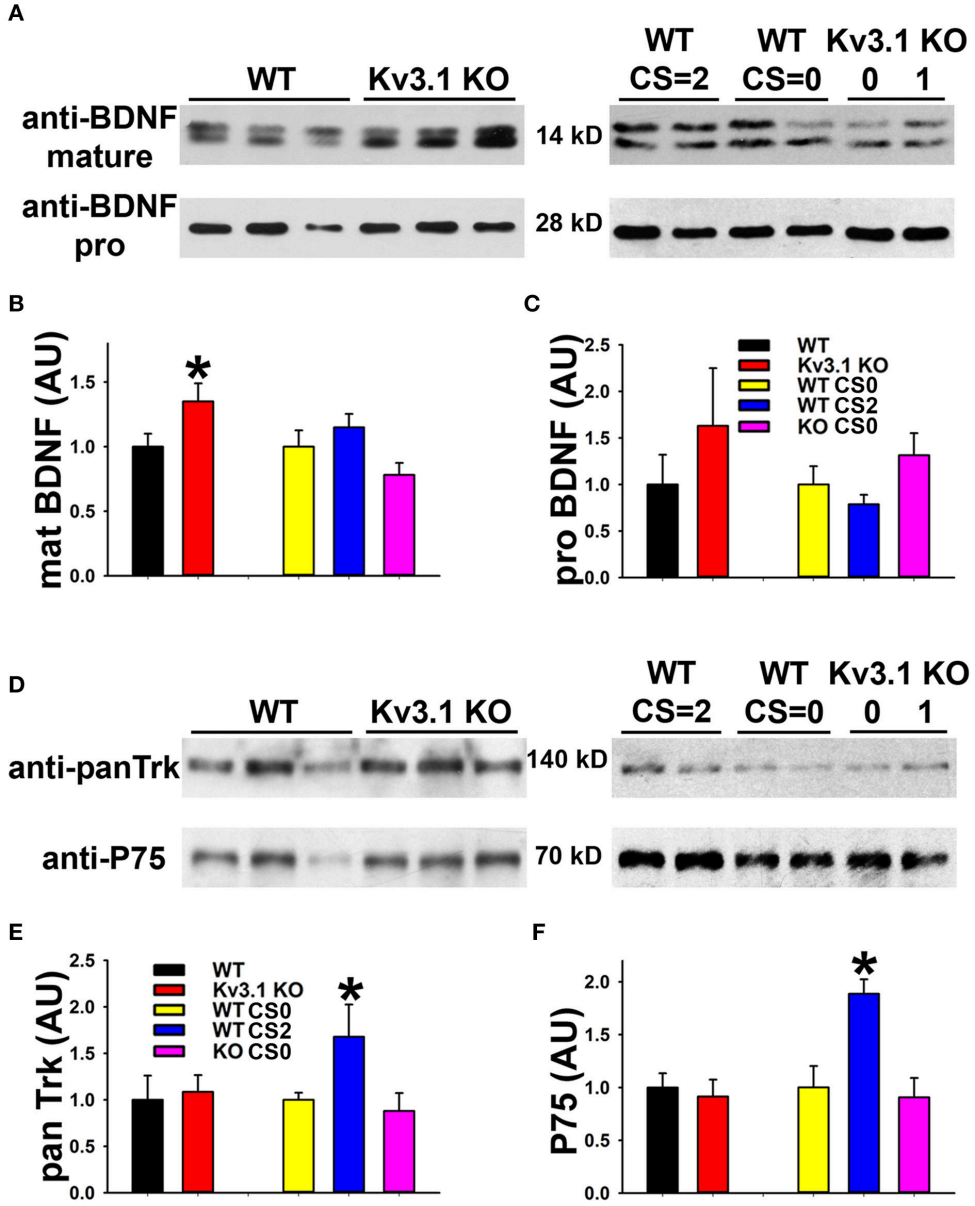
Biochemical analysis of BDNF and its receptor levels in Kv3.1 KO and EAE mice. **(A)** Western blot analysis of alterations of mature BDNF and pro-BDNF expression levels in Kv3.1 KO mice and at different stages of EAE. **(B)** Summary of mature BDNF levels. **(C)** Summary of pro-BDNF levels. **(D)** Western blot analysis of alterations of pan-Trk and P75 expression levels in Kv3.1 KO mice and at different stages of EAE. **(E)** Summary of pan-Trk levels. **(F)** Summary of P75 levels. Unpaired Student *t*–test between WT and Kv3.1 KO mice: ^*^p < 0.05. One-way ANOVA followed by Dunnett's test in EAE mice. ^*^p < 0.05.

We next examined the expression of BDNF and its receptors, Tyrosine Receptor Kinase B (TrkB) in EAE. The BDNF expression level in SCWM astrocytes markedly increased at the peak stage of EAE and reduced, but remained higher than the baseline, at the late stage (Figure 5F). To determine potential activation of BDNF receptors, we examined the levels of phosphorylated (activated) TrkB receptor using an anti-phospho(Y816)-TrkB (anti-TrkB-p) antibody in SCWM. At peak EAE, signals for TrkB-p increased along some axonal segments surrounding the lesion sites (Figures 5C,D and Movies S7, S8), similar to the staining for Kv3.1 and Kv3.3. To verify the immunostaining results, we analyzed global changes of related signaling components by Western blotting. Mature BDNF did increase in Kv3.1 KO mice, and also markedly increased in EAE, whereas no change was observed for pro-BDNF (Figures 6A–C). Another receptor of BDNF, P75, did not change when Kv3.1 was deleted, but significantly increased during EAE (Figures 6D–F). No change was observed for the total pan-Trk level for either Kv3.1 KO or EAE (Figures 6D–F). It is important to note that altered expression in individual cells may or may not correlate with global changes. Nonetheless, our results indicate a key role of BDNF in mediating the effect of Kv3 channels during inflammatory demyelination and axonal degeneration.

### Alterations of Kv3 channels, BDNF and activated TrkB in post-mortem brains of MS patients

**Figure 7:**
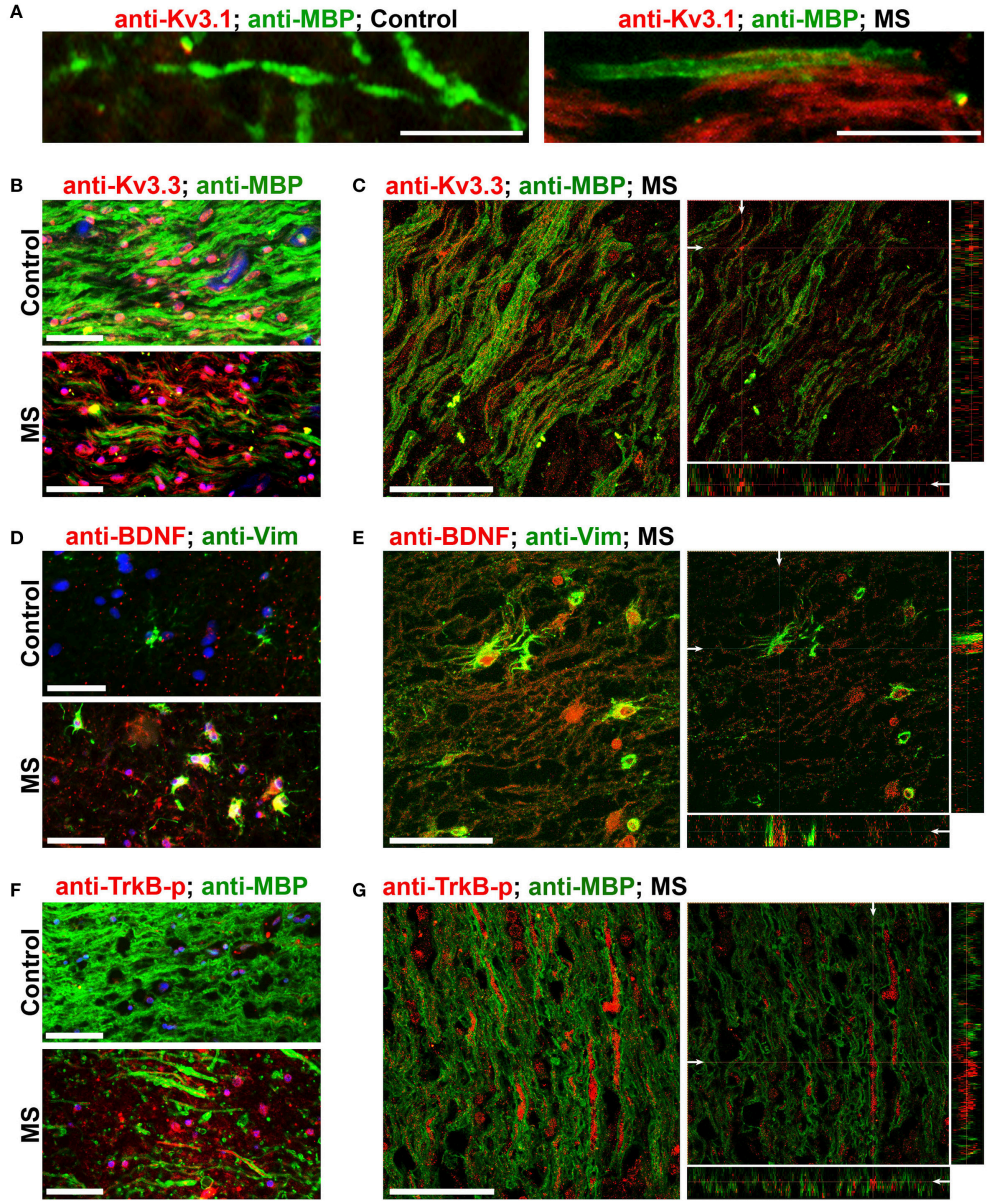
Alterations of Kv3 channels and BDNF in post-mortem tissues of MS patients. **(A)** Costaining of post-mortem brain sections from a non-MS individual (control) and an MS patient for Kv3.1 (red) and MBP (green). **(B)** Post-mortem brain sections from a control individual (top) and an MS patient (bottom) were costained for Kv3.3 channels (red) and MBP (green). **(C)** A 3-D confocal image stack for Kv3.3 (red) and MBP (green) in an MS lesion. **(D)** Sections from post-mortem brains were costained for Vim (green) and BDNF (red). **(E)** A 3-D confocal image stack in an MS lesion from **(D)**. **(F)** Sections from post-mortem brains were costained for phospho-TrkB (TrkB-p, red) and MBP (green). **(G)** A 3-D confocal image stack in an MS lesion **(F)**. Scale bars, 50 μm.

To verify whether our key findings in the EAE studies are relevant to MS, we performed immunofluorescence staining on post-mortem brain sections from non-MS subjects (as controls) and MS patients containing active or chronic silent lesions. Surrounding lesion areas, there were also demyelinated axonal segments with upregulated Kv3.1 and Kv3.3 channels (Figures 7A–C and Table 1). Compared to control, the levels of both Vim and BDNF increased in astrocyte-like cells around lesion edges (Figures 7D,E and Table 1), consistent with the EAE results (Figure 4). Moreover, TrkB-p levels were also elevated in axonal segments in lesion areas (Figures 7F,G). Taken together, our results show that surrounding and within lesion sites of both MS and EAE, activated astrocytes express BDNF, while some demyelinated axons contain higher levels of Kv3.1/Kv3.3 and activated TrkB receptors.

**Table 1:** Comparison of post-mortem protein expression in MS patients and non-MS control individuals.

**Case ID**	**Age**	**Sex**	**Disease course**	**Disease duration**	**Cause of death**	**Post-mortem interval**	**Axonal protein expression**			**Astrocyte protein expression**		**Loss of MBP**
							**Kv3.1b**	**Kv3.3**	**TrkB-p**	**Vim**	**BDNF**	
18B	52	F	N/A	N/A	Myocardial infarction	10 hrs	+	−	−	+	+	−
15B	80	M	N/A	N/A	Unknown	21 hrs	+	+	+	++	+	−
24B	41	M	N/A	N/A	Metastatic cancer	12 hrs	+	+	+	+	+	−
18B-TC	52	F	N/A	N/A	Myocardial infarction	10 hrs	+	+	−	+	+	−
X3818E	59	F	SPMS	25 yrs	Bronchopneumonia	12 hrs	++	+	+++	+	+++	+++
X4103-G	36	F	RRMS	11 yrs	Sepsis	5 hrs	++	++	+++	++++	+++	+++
X2993	32	M	RRMS	10 yrs	Pneumonia	1.5 hrs	+	++	++++	++++	++	+++
Ax83165-II	32	F	RRMS	6 yrs	Unknown	8 hrs	+	+	++	++++	+++	+++
XC2985-2	50	F	SPMS	10 yrs	Respiratory failure	6.5 hrs	++	++	++++	+++	++	++++

*SPMS, Secondary progressive multiple sclerosis; RRMS, relapsing remitting multiple sclerosis. Age in years. yrs, years; hrs, hours; TrkB-p, phosphorylated tyrosine receptor kinase; Vim, Vimentin; BDNF, brain derived neurotrophic factor; MBP, myelin base protein; “–“ negative; “+” positive. All patients were de-identified*.

### BDNF treatment promotes myelination and alters action potential firing in SC myelin coculture

**Figure 8:**
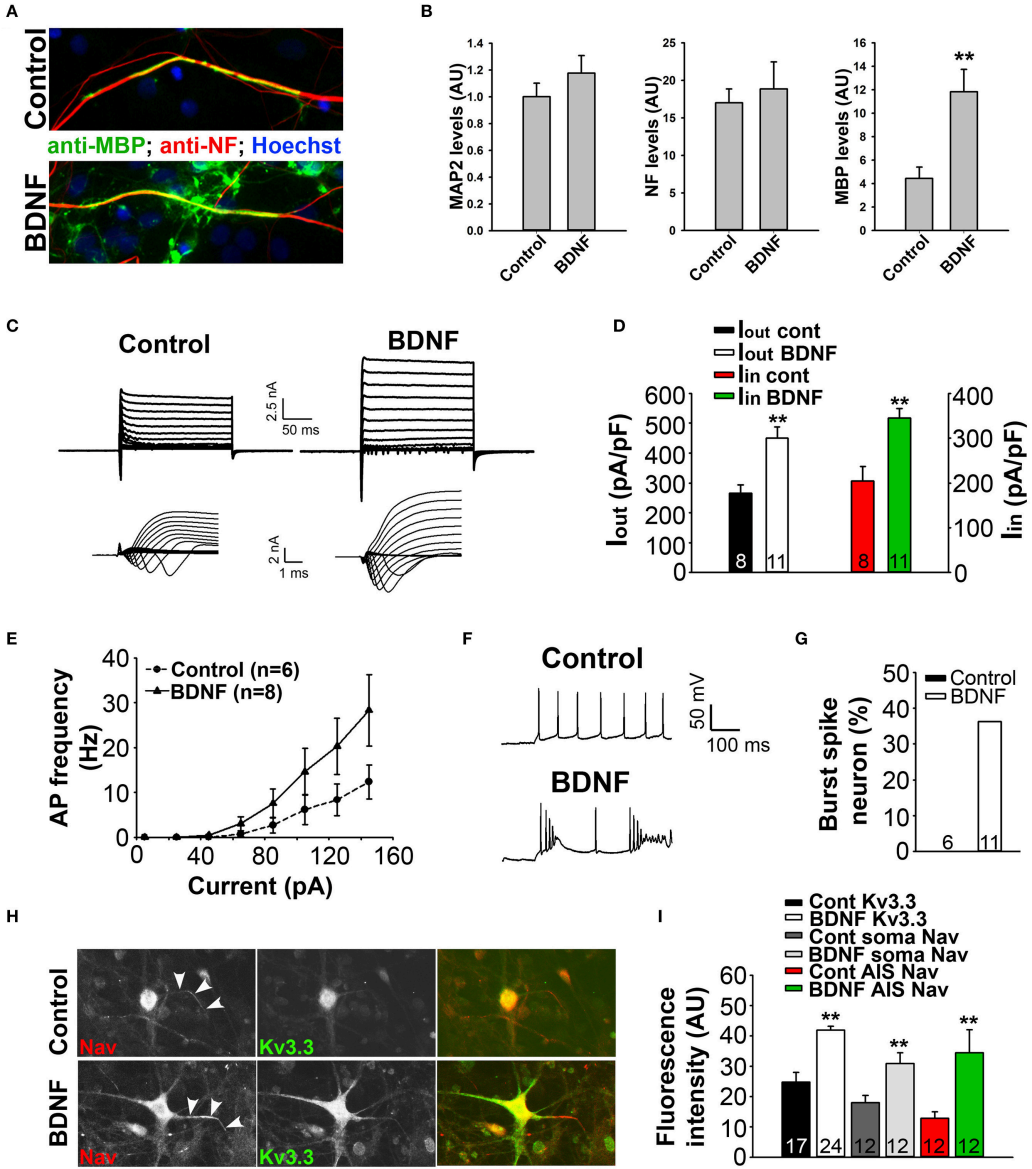
BDNF treatment stimulates axon myelination and increases neuronal excitability. **(A)** BDNF treatment (bottom) increased axon myelination of cultured SC neurons. **(B)** Summary of BDNF treatment on the levels of MAP2, neurofilament (NF) and MBP. **(C)** BDNF treatment increased the levels of inward and outward currents under the voltage-clamp mode. **(D)** Summary of inward and outward current amplitudes. **(E)** BDNF treatment increased action potential firing frequency revealed by the input-output relationship. **(F,G)** BDNF treatment increased burst firing. **(H)** BDNF treatment (bottom) increased the endogenous expression levels of Kv3.3 (green) and Nav (red) channels. **(I)** Summary of the expression levels of endogenous Kv3.3 and Nav channels at the soma and AIS. Unpaired Student *t*–test: ^**^p < 0.01. Scale bars, 50 μm.

To determine the mechanism underlying BDNF signaling in myelination, axonal degeneration, and neuronal survival in EAE and MS, we examined the effect of BDNF treatment on myelin coculture of SC neurons. To carry out this experiment, we first developed a myelin coculture method for SC neurons based on the method that we published for myelin coculture of hippocampal neurons (Gardner et al., 2012). The SC myelin coculture, however, required an astrocyte layer and mixed neurons and glia from SC at the embryonic stage. BDNF treatment (for 1 week during myelination) significantly enhanced myelin formation by increasing the expression of MBP, but did not change neuronal dendrites and axons indicated by microtubule associated protein 2 (MAP2) and neurofilament staining, respectively (Figures 8A,B). This result indicates that BDNF treatment under this condition stimulated myelin formation, but did not alter neuronal and axonal survival.

To determine how BDNF treatment may affect neuronal excitability, we performed whole-cell recording under both voltage- and current-clamp modes. Cultured SC neurons with large soma, likely the motor neurons, were recorded under control and treated conditions. Interestingly, the treatment markedly increased both inward and outward currents, which were most likely mediated by Nav and Kv channels, respectively (Figures 8C,D). Consistent with this result, the treatment also increased firing frequency and burst firing of action potentials (Figures 8E–G). Remarkably, immunostaining results showed that the endogenous levels of Kv3.3 and pan-Nav channels were significantly increased at the soma, as well as at the axon initial segments (Figures 8H,I). Therefore, the staining data are consistent with the electrophysiological results, indicating that BDNF treatment increases neuronal excitability through increasing the expression of Nav and Kv3.3 channels.

## Discussion

**Figure 9:**
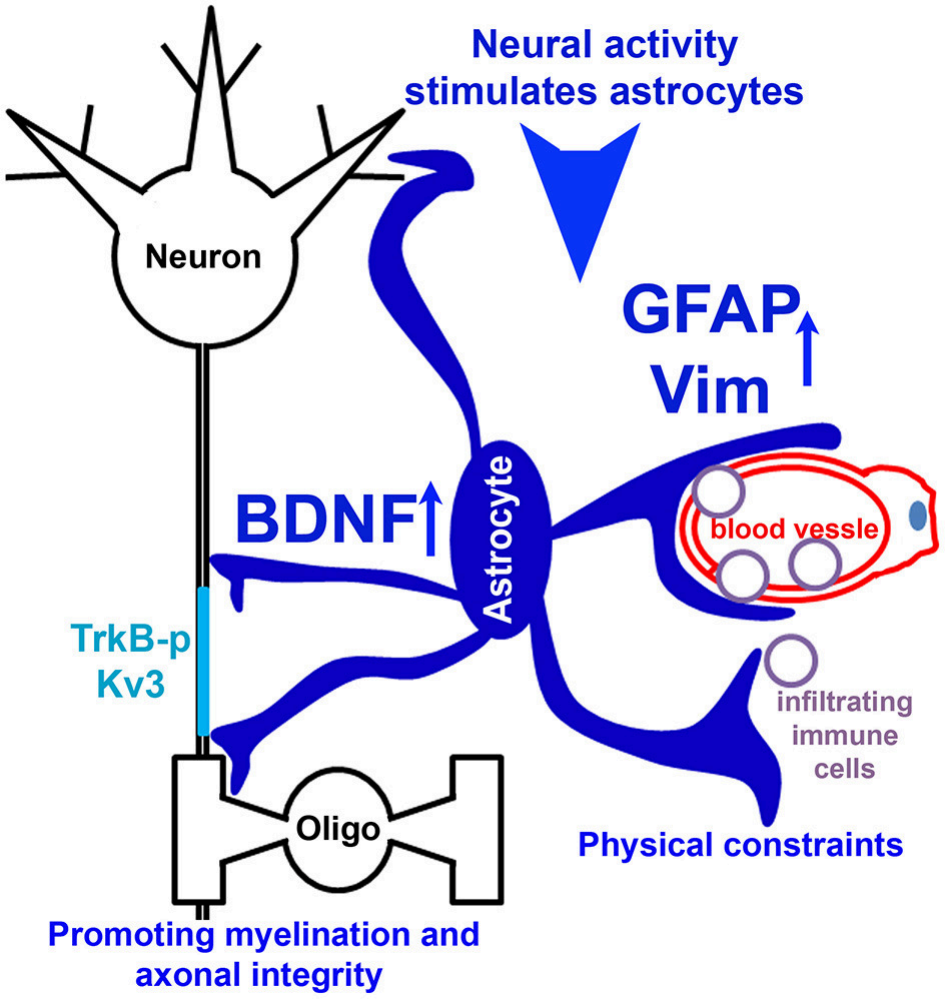
Hypothetical model diagram of the role of Kv3 channels in lesion formation in EAE and MS. Neural activity stimulates radial astrocytes, which upregulate the expression of GFAP and Vim, as well as BDNF. Upregulated intermediate filaments (GFAP and Vim) increase the rigidness of astrocytes, thereby deterring migration and proliferation of infiltrating immune cells to limit lesion formation. On the other hand, BDNF signaling promotes axon remyelination and prevents axonal degeneration, and thus contributes to lesion repair.

In the present study, the novel finding that reduced EAE severity in Kv3.1 KO mice correlates with reduced lesion number and size prompted us to pursue the underlying mechanism using both *in vivo* and *in vitro* systems. Our experimental results indicate the following two potential mechanisms (Figure 9). (1) Upregulated intermediate filaments (GFAP and Vim) increase the rigidness of astrocytes in Kv3.1 KO mice and hence can deter migration and proliferation of infiltrating immune cells. (2) Through activation of BDNF signaling in activated radial astroglia in Kv3.1 KO mice, lesion formation can be deterred and lesions may even be repaired. Taken together, our study has provided new mechanistic insights into the beneficial effects of Kv channel suppression in treating EAE/MS. This may lead to a new model for activity-dependent prevention and repair of inflammatory axonal lesions.

4-AP is used as a symptomatic treatment of decreased walking capacity in MS patients. It also has beneficial effects on cognition, upper extremity function and bowel and bladder function. Slow release 4-AP has only mild to moderate side effects in some patients, including paresthesia, dizziness, nausea/vomiting, falls/balance disorders, insomnia, urinary tract infections, and asthenia (Jensen et al., 2014). Long-term usage of 4-AP (9–12 months) appears to be safe and leads to greater improvement in the Paced Auditory Serial Addition Test (a test of cognitive function), compared to short-term usage (2 weeks) (Cameron et al., 2014; Ruck et al., 2014). A systematic study will be needed to determine whether and how the long-term usage of 4-AP affects MS lesions. On the other hand, a recent study showed that 4-AP treatment at the beginning of clinical signs in MOG-induced chronic EAE did not significantly alter EAE severity and pathology (Gobel et al., 2013), different from our studies with Kv3.1 KO mice. However, the 4-AP treatment did significantly improve the mobility of the EAE mice (Gobel et al., 2013), consistent with our findings. It is important to note that 4-AP blocks a number of important Kv channels, the lack of clear beneficial effects of 4-AP on EAE may result from mixed positive and negative actions by different Kv channels. Nonetheless, it is possible that specifically blocking the activity of Kv3.1 channel provides a long-term beneficial effect on MS progression.

Kv3.1 is likely a main target for mediating the effects of 4-AP on MS progression. Kv3.1 IC_50_ for 4-AP is around 29 μM, the lowest among all Kv channels (Judge and Bever, 2006). The IC_50_s for Kv1.1, Kv1.2, and Kv1.3 are around 290, 590, and 195 μM, respectively. The IC_50_s for Kv2 and Kv4 channels are over 1 mM (Judge and Bever, 2006). Recently, it was reported that 4-AP may enhance synaptic transmission by stimulating the activity of L-type Cav channels (Wu et al., 2009; Li et al., 2012). 4-AP started to activate L-type Cav channels at 100 μM, but had no effect at 30 μM (Li et al., 2012). Therefore, Kv3.1 channels display the highest sensitivity to 4-AP among all known 4-AP targets. However, despite the physiological significance of Kv3.1, its role in MS or EAE progression was not previously investigated.

Kv3.1 KO mice are viable and relatively healthy, which allows us to perform EAE with them. We found that EAE signs were significantly reduced in Kv3.1 KO mice at both peak and late stages (Figure 1). Anatomical analysis revealed that reduced EAE severity in Kv3.1 KO mice correlated with lesion reduction in size and number (Figure 1). Such reduction is consistent with preserved neurophysiological functions (e.g., mobility) of Kv3.1 KO mice. Our results indicate that deleting Kv3.1 may hinder EAE lesion formation and perhaps even promote lesion repair, which we will discuss in the following sections. Furthermore, we identified a biased formation of EAE lesions in the dorsal WM of SC, compared with the ventral WM (Figure 1). The mechanism underlying this biased lesion formation in chronic EAE is an interesting topic for future investigation.

In the present study, we used Kv3.1 KO mice allowing us to analyze the effect of specific deletion of a single Kv channel on EAE severity. It is important to note that this strategy differs from the 4-AP treatment of EAE and MS. The 4-AP treatment likely acts through multiple targets, causing mixed effects. Our finding that Kv3.1 deletion lessens EAE severity has raised an interesting question for future investigation. Is it possible that a Kv3.1-specific blocker can be more effective than 4-AP in treating MS and have a long-term beneficial effect on MS patients? Currently, there is no highly specific blocker for Kv3.1 channels.

The effect of Kv3.1 deletion on EAE severity is mainly through the nervous system, but not the immune system. Kv3.1 does not appear to play a major role in activated immune cells. Kv3.1 current was only implicated in double-negative and CD8^+^ T cells in mice (Chandy et al., 1990), but not in humans (Cahalan and Chandy, 2009). Our experiments using adoptive transfer EAE showed that deleting Kv3.1 does not affect the competence of the immune cells to induce EAE when being transferred into naïve WT mice. Immune cells with or without Kv3.1 also appeared to secrete various cytokines at similar levels (Figure 2). On the other hand, both Kv3.1 and Kv3.3, especially Kv3.3, were upregulated in some injured axons in EAE and MS lesions (Figures 3, 7). It is important to note that this localized upregulation differs from global changes and its exact impact on axonal functions remains to be determined. Although Kv3.1 and Kv3.3 can assemble into heterotetramers, they still have different biophysical and pharmacological features with different expression and localization patterns. Nonetheless, upregulated Kv3.3 likely works together with other ion channels to alter axonal functions, such as several Ca^2+^- and Na^+^-permeable channels that were also upregulated in injured axons in EAE and MS lesions (Kornek et al., 2001; Craner et al., 2004; Vergo et al., 2011; Schattling et al., 2012). Downstream of the channel alteration, the intra-axonal Ca^2+^ level, known to be involved in axonal degeneration, may play a key role here. Therefore, potential coordinated actions of these ion channels may determine the fate of the subset of axons in EAE and MS lesions, recovery or degeneration.

How does Kv3.1 deletion reduce EAE lesion formation? Activated astrocytes may be the key mediator. Our previous study showed that Bergmann radial glia in the cerebellum appeared activated in Kv3.1 KO mice (Jukkola et al., 2013). In the present study, we found that astrocytes in SC of Kv3.1 KO mice were activated with upregulation of intermediate filaments, GFAP and Vim (Figure 4). In particular, Vim was significantly upregulated in SC radial astroglia of KO mice (Figure 4). Activation of astrocytes observed in Kv3.1 KO mice may be triggered by altered activity of related neural circuits, in which Kv3.1 deletion may directly increase principal neuron excitability or reduce fast spiking of some GABAergic interneurons (Rudy and McBain, 2001). Importantly, upregulation of GFAP and Vim in astrocytes in Kv3.1 KO mice may increase rigidness of these glial cells and hence deter migration and proliferation of infiltrating immune cells in EAE. GFAP and Vim KO mice develop more severe EAE than WT mice (Liedtke et al., 1998). The physical properties of astrocytes are very important to their function in maintaining and regulating sensorimotor circuits. Although it is accepted that astrocytes are involved in EAE and MS lesions (Jukkola et al., 2013), little is known about a potential role of radial astroglia in the pathogenic processes. Given that radial glia play critical roles in cytogenesis, patterning and boundary formation in the developing SC (McDermott et al., 2005), it is really important to identify the relevance of these cells to the inflammatory demyelinating lesions.

Released factors from activated radial astroglial cells may regulate lesion formation in EAE and MS. Our results show that Kv3.1 deletion activates radial astroglia with increases Vim and BDNF expression (Figures 4–7), while BDNF may also mediate the partial recovery often observed in chronic EAE. BDNF signaling was reported to promote myelination, protect neurons, and promote neuronal differentiation. In EAE and MS lesions, we indeed observed upregulation of activated TrkB receptors in a subset of axons, in addition to astrocytic BDNF (Figures 5, 7). These axons with activated TrkB receptors may be those that can survive and even be remyelinated during the development of lesions. Previous studies showed that astrocytic BDNF promotes remyelination via TrkB in the cuprizone model (Fulmer et al., 2014). The reduced EAE severity and lesions at the peak stage in Kv3.1 KO mice indicate that Kv3.1 deletion likely inhibits lesion formation. At the late EAE stage, Kv3.1 KO mice did display milder EAE severity than their WT littermates (Figure 1), suggesting a potential repair mechanism. This repair may in part share the same signaling pathway with the spontaneous repair in chronic EAE. Increased BDNF levels were observed in Vim-positive astroglia in EAE lesions at the peak stage in both WT and Kv3.1 KO mice (Figure 5F). However, since lesion development and repair are likely overlapping events in terms of time course, currently we cannot distinguish between the two possibilities, prevention/repair vs. repair alone, in our studies.

To further understand the mechanism by which BDNF may regulate inflammatory demyelinating lesions, we developed a new myelin coculture system containing SC neurons and oligodendrocytes. BDNF treatment of the coculture was carried out at the myelinating stage, after SC neurons had fully differentiated and oligodendrocytes proliferated. Our results show that BDNF treatment promoted myelination, but did not affect neuron survival and differentiation (Figure 8), consistent with our hypothesis based on the EAE data. Furthermore, using whole-cell recording and immunostaining, we surprisingly found that the BDNF treatment promoted the expression of Kv3.3 and Nav channels (Figure 8). As a result, the waveform of action potentials contained bursting (Figure 8), which is an indication of Kv3.3 activation in action potential waveforms of cerebellar Purkinje neurons, although it is still unknown whether Kv3.3 induces bursting in other neurons as well. Taken together, whether and how activated TrkB receptors in axons stimulate the expression of Kv3.3 and/or other Ca^2+^-permeable channels remain to be determined in future studies.

Our data support a model that both voluntary and involuntary nerve activation is beneficial for lesion reduction and repair in inflammatory demyelinating diseases, including MS. At the same time, our studies have also raised some very interesting questions for future investigation. Kv3.1 is the most sensitive target of 4-AP, which increases nerve conduction involuntarily. Interestingly, 4-AP treatment is beneficial not only to MS patients, but also to patients suffering from spinal cord injury and cerebellar disorders (Segal et al., 1999; Kalla et al., 2007). On the other hand, voluntary exercise is known to increase BDNF levels (Ying et al., 2005; Ding et al., 2011), and is currently believed to be beneficial for MS patients. Taken together, further investigation along this research line may contribute to the development of a new therapeutic strategy of activity-dependent lesion reduction and repair in inflammatory neurodegenerative diseases.

## Author contributions

CG designed and supervised the research; PJ, YG, and CG performed experiments, analyzed data, and made figures. CG, AL, and PJ wrote and revised the paper.

### Conflict of interest statement

The authors declare that the research was conducted in the absence of any commercial or financial relationships that could be construed as a potential conflict of interest.

## Supplementary material

Movie S1 An image stack from a longitudinal section of SCWM in a control mouse. FMG is in green and anti-Kv3.3 staining in red. This is the supplementary movie for Figure 3G upper panel.

Movie S2 An image stack from an EAE lesion in a longitudinal section of SCWM in an EAE mouse. FMG is in green and anti-Kv3.3 staining in red. This is the supplementary movie for Figure 3G lower panel.

Movie S3 An image stack from a cross section of ventral SCWM in a WT control mouse. The anti-GFAP staining is in green and anti-Vim staining in red. This is the supplementary movie for Figure 4A.

Movie S4 An image stack from a cross section of ventral SCWM in a Kv3.1 KO mouse. The anti-GFAP staining is in green and anti-Vim staining in red. This is the supplementary movie for Figure 4B.

Movie S5 An image stack from a cross section of ventral SCWM in a WT control mouse. The anti-BDNF staining is in red and anti-Vim staining in green. This is the supplementary movie for Figure 5A.

Movie S6 An image stack from a cross section of ventral SCWM in a Kv3.1 KO mouse. The anti-BDNF staining is in red and anti-Vim staining in green. This is the supplemental movie for Figure 5B.

Movie S7 An image stack from a longitudinal section of SCWM in a WT control mouse. The anti-Vim staining is in green and anti-TrkB-p staining in red. This is the supplementary movie for Figure 5C.

Movie S8 An image stack from an EAE lesion in a longitudinal section of SCWM in an EAE mouse at the peak stage. The anti-Vim staining is in green and anti-TrkB-p staining in red. This is the supplementary movie for Figure 5D.

## References

[B1] Barry J. Xu M. Gu Y. Dangel A. Jukkola P. Shrestha C.. (2013). Activation of conventional kinesin motors in clusters by Shaw voltage-gated K+ channels. J. Cell Sci. 126, 2027–2041. 10.1242/jcs.122234 23487040 PMC3666255

[B2] Brew H. M. Gittelman J. X. Silverstein R. S. Hanks T. D. Demas V. P. Robinson L. C.. (2007). Seizures and reduced life span in mice lacking the potassium channel subunit Kv1.2, but hypoexcitability and enlarged Kv1 currents in auditory neurons. J. Neurophysiol. 98, 1501–1525. 10.1152/jn.00640.2006 17634333

[B3] Cahalan M. D. Chandy K. G. (2009). The functional network of ion channels in T lymphocytes. Immunol. Rev. 231, 59–87. 10.1111/j.1600-065X.2009.00816.x 19754890 PMC3133616

[B4] Cameron M. H. Fitzpatrick M. Overs S. Murchison C. Manning J. Whitham R. (2014). Dalfampridine improves walking speed, walking endurance, and community participation in veterans with multiple sclerosis: a longitudinal cohort study. Mult. Scler. 20, 733–738. 10.1177/1352458513507356 24099749

[B5] Chan J. R. Watkins T. A. Cosgaya J. M. Zhang C. Chen L. Reichardt L. F. (2004). NGF controls axonal receptivity to myelination by Schwann cells or oligodendrocytes. Neuron 43, 183–191. 10.1016/j.neuron.2004.06.024 15260955 PMC2758239

[B6] Chandy K. G. Cahalan M. D. Grissmer S. (1990). Autoimmune diseases linked to abnormal K+ channel expression in double-negative CD4-CD8- T cells. Eur. J. Immunol. 20, 747–751. 10.1002/eji.1830200406 1971790

[B7] Chandy K. G. Wulff H. Beeton C. Pennington M. Gutman G. A. Cahalan M. D. (2004). K+ channels as targets for specific immunomodulation. Trends Pharmacol. Sci. 25, 280–289. 10.1016/j.tips.2004.03.010 15120495 PMC2749963

[B8] Chang S. Y. Zagha E. Kwon E. S. Ozaita A. Bobik M. Martone M. E.. (2007). Distribution of Kv3.3 potassium channel subunits in distinct neuronal populations of mouse brain. J. Comp. Neurol. 502, 953–972. 10.1002/cne.21353 17444489

[B9] Craner M. J. Newcombe J. Black J. A. Hartle C. Cuzner M. L. Waxman S. G. (2004). Molecular changes in neurons in multiple sclerosis: altered axonal expression of Nav1.2 and Nav1.6 sodium channels and Na+/Ca2+ exchanger. Proc. Natl. Acad. Sci. U.S.A. 101, 8168–8173. 10.1073/pnas.0402765101 15148385 PMC419575

[B10] Davis F. A. Stefoski D. Rush J. (1990). Orally administered 4-aminopyridine improves clinical signs in multiple sclerosis. Ann. Neurol. 27, 186–192. 10.1002/ana.410270215 2317014

[B11] Debanne D. (2004). Information processing in the axon. Nat. Rev. Neurosci. 5, 304–316. 10.1038/nrn1397 15034555

[B12] Ding Q. Ying Z. Gomez-Pinilla F. (2011). Exercise influences hippocampal plasticity by modulating brain-derived neurotrophic factor processing. Neuroscience 192, 773–780. 10.1016/j.neuroscience.2011.06.032 21756980 PMC3225196

[B13] Dutta R. Trapp B. D. (2011). Mechanisms of neuronal dysfunction and degeneration in multiple sclerosis. Prog. Neurobiol. 93, 1–12. 10.1016/j.pneurobio.2010.09.005 20946934 PMC3030928

[B14] Espejo C. Montalban X. (2012). Dalfampridine in multiple sclerosis: from symptomatic treatment to immunomodulation. Clin. Immunol. 142, 84–92. 10.1016/j.clim.2011.06.004 21742559

[B15] Evers P. Uylings H. B. (1997). An optimal antigen retrieval method suitable for different antibodies on human brain tissue stored for several years in formaldehyde fixative. J. Neurosci. Methods 72, 197–207. 10.1016/S0165-0270(96)02204-2 9133585

[B16] Ferguson B. Matyszak M. K. Esiri M. M. Perry V. H. (1997). Axonal damage in acute multiple sclerosis lesions. Brain 3, 393–399. 10.1093/brain/120.3.393 9126051

[B17] Friese M. A. Craner M. J. Etzensperger R. Vergo S. Wemmie J. A. Welsh M. J.. (2007). Acid-sensing ion channel-1 contributes to axonal degeneration in autoimmune inflammation of the central nervous system. Nat. Med. 13, 1483–1489. 10.1038/nm1668 17994101

[B18] Fulmer C. G. VonDran M. W. Stillman A. A. Huang Y. Hempstead B. L. Dreyfus C. F. (2014). Astrocyte-derived BDNF supports myelin protein synthesis after cuprizone-induced demyelination. J. Neurosci. 34, 8186–8196. 10.1523/JNEUROSCI.4267-13.2014 24920623 PMC4051974

[B19] Gardner A. Jukkola P. Gu C. (2012). Myelination of rodent hippocampal neurons in culture. Nat. Protoc. 7, 1774–1782. 10.1038/nprot.2012.100 22955693 PMC3536533

[B20] Gobel K. Wedell J. H. Herrmann A. M. Wachsmuth L. Pankratz S. Bittner S. (2013). 4-Aminopyridine ameliorates mobility but not disease course in an animal model of multiple sclerosis. Exp. Neurol. 248, 62–71. 10.1016/j.expneurol.2013.05.016 23748135

[B21] Gocke A. R. Lebson L. A. Grishkan I. V. Hu L. Nguyen H. M. Whartenby K. A.. (2012). Kv1.3 deletion biases T cells toward an immunoregulatory phenotype and renders mice resistant to autoimmune encephalomyelitis. J. Immunol. 188, 5877–5886. 10.4049/jimmunol.1103095 22581856 PMC3370138

[B22] Goodman A. D. Brown T. R. Krupp L. B. Schapiro R. T. Schwid S. R. Cohen R.. (2009). Sustained-release oral fampridine in multiple sclerosis: a randomised, double-blind, controlled trial. Lancet 373, 732–738. 10.1016/S0140-6736(09)60442-6 19249634

[B23] Gu C. Barry J. (2011). Function and mechanism of axonal targeting of voltage-sensitive potassium channels. Prog. Neurobiol. 94, 115–132. 10.1016/j.pneurobio.2011.04.009 21530607 PMC3112463

[B24] Gu C. Gu Y. (2011). Clustering and activity tuning of kv1 channels in myelinated hippocampal axons. J. Biol. Chem. 286, 25835–25847. 10.1074/jbc.M111.219113 21602278 PMC3138291

[B25] Gu C. Zhou W. Puthenveedu M. A. Xu M. Jan Y. N. Jan L. Y. (2006). The microtubule plus-end tracking protein EB1 is required for Kv1 voltage-gated K+ channel axonal targeting. Neuron 52, 803–816. 10.1016/j.neuron.2006.10.022 17145502

[B26] Gu Y. Barry J. Gu C. (2013). Kv3 channel assembly, trafficking and activity are regulated by zinc through different binding sites. J. Physiol. 591, 2475–2490. 10.1113/jphysiol.2013.251983 23420657 PMC3678039

[B27] Gu Y. Barry J. McDougel R. Terman D. Gu C. (2012). Alternative splicing regulates Kv3.1 polarized targeting to adjust the maximal spiking frequency. J. Biol. Chem. 287, 1755–1769. 10.1074/jbc.M111.299305 22105078 PMC3265858

[B28] Hayes K. C. (2004). The use of 4-aminopyridine (fampridine) in demyelinating disorders. CNS Drug Rev. 10, 295–316. 10.1111/j.1527-3458.2004.tb00029.x 15592580 PMC6741729

[B29] Hayes K. C. Katz M. A. Devane J. G. Hsieh J. T. Wolfe D. L. Potter P. J.. (2003). Pharmacokinetics of an immediate-release oral formulation of Fampridine (4-aminopyridine) in normal subjects and patients with spinal cord injury. J. Clin. Pharmacol. 43, 379–385. 10.1177/0091270003251388 12723458

[B30] Hille B. (2001). Ion Channels of Excitable Membranes. Sunderland, MA: Sinauer.

[B31] Ho C. S. Grange R. W. Joho R. H. (1997). Pleiotropic effects of a disrupted K+ channel gene: reduced body weight, impaired motor skill and muscle contraction, but no seizures. Proc. Natl. Acad. Sci. U.S.A. 94, 1533–1538. 10.1073/pnas.94.4.1533 9037088 PMC19826

[B32] Huang E. J. Reichardt L. F. (2001). Neurotrophins: roles in neuronal development and function. Annu. Rev. Neurosci. 24, 677–736. 10.1146/annurev.neuro.24.1.677 11520916 PMC2758233

[B33] Hurlock E. C. Bose M. Pierce G. Joho R. H. (2009). Rescue of motor coordination by Purkinje cell-targeted restoration of Kv3.3 channels in Kcnc3-null mice requires Kcnc1. J. Neurosci. 29, 15735–15744. 10.1523/JNEUROSCI.4048-09.2009 20016089 PMC3849660

[B34] Hurlock E. C. McMahon A. Joho R. H. (2008). Purkinje-cell-restricted restoration of Kv3.3 function restores complex spikes and rescues motor coordination in Kcnc3 mutants. J. Neurosci. 28, 4640–4648. 10.1523/JNEUROSCI.5486-07.2008 18448641 PMC6670432

[B35] Huss D. J. Winger R. C. Peng H. Yang Y. Racke M. K. Lovett-Racke A. E. (2013). TGF-beta enhances effector Th1 cell activation but promotes self-regulation via IL-10. J. Immunol. 184, 5628–5636. 10.4049/jimmunol.1000288 20393141 PMC3804066

[B36] Jensen H. B. Ravnborg M. Dalgas U. Stenager E. (2014). 4-Aminopyridine for symptomatic treatment of multiple sclerosis: a systematic review. Ther. Adv. Neurol. Disord. 7, 97–113. 10.1177/1756285613512712 24587826 PMC3932769

[B37] Joho R. H. Street C. Matsushita S. Knopfel T. (2006). Behavioral motor dysfunction in Kv3-type potassium channel-deficient mice. Genes Brain Behav. 5, 472–482. 10.1111/j.1601-183X.2005.00184.x 16923152

[B38] Judge S. I. Bever C. T. Jr. (2006). Potassium channel blockers in multiple sclerosis: neuronal Kv channels and effects of symptomatic treatment. Pharmacol. Ther. 111, 224–259. 10.1016/j.pharmthera.2005.10.006 16472864

[B39] Jukkola P. Guerrero T. Gray V. Gu C. (2013). Astrocytes differentially respond to inflammatory autoimmune insults and imbalances of neural activity Acta Neuropathol. Commun. 1:70. 10.1186/2051-5960-1-70 24252623 PMC3893391

[B40] Jukkola P. Lovett-Racke A. Zamvil S. S. Gu C. (2012). K^+^ channel alterations in the progression of experimental autoimmune encephalomyelitis. Neurobiol. Dis. 47, 280–293. 10.1016/j.nbd.2012.04.012 22560931 PMC3367054

[B41] Kalla R. Glasauer S. Buttner U. Brandt T. Strupp M. (2007). 4-aminopyridine restores vertical and horizontal neural integrator function in downbeat nystagmus. Brain 130, 2441–2451. 10.1093/brain/awm172 17664175

[B42] Kerschensteiner M. Gallmeier E. Behrens L. Leal V. V. Misgeld T. Klinkert W. E.. (1999). Activated human T cells, B cells, and monocytes produce brain-derived neurotrophic factor *in vitro* and in inflammatory brain lesions: a neuroprotective role of inflammation? J. Exp. Med. 189, 865–870. 10.1084/jem.189.5.865 10049950 PMC2192942

[B43] Kornek B. Storch M. K. Bauer J. Djamshidian A. Weissert R. Wallstroem E.. (2001). Distribution of a calcium channel subunit in dystrophic axons in multiple sclerosis and experimental autoimmune encephalomyelitis. Brain 124, 1114–1124. 10.1093/brain/124.6.1114 11353727

[B44] Kriegstein A. Alvarez-Buylla A. (2009). The glial nature of embryonic and adult neural stem cells. Annu. Rev. Neurosci. 32, 149–184. 10.1146/annurev.neuro.051508.135600 19555289 PMC3086722

[B45] Kutzelnigg A. Lucchinetti C. F. Stadelmann C. Bruck W. Rauschka H. Bergmann M.. (2005). Cortical demyelination and diffuse white matter injury in multiple sclerosis. Brain 128, 2705–2712. 10.1093/brain/awh641 16230320

[B46] Lee D. H. Geyer E. Flach A. C. Jung K. Gold R. Flugel A.. (2012). Central nervous system rather than immune cell-derived BDNF mediates axonal protective effects early in autoimmune demyelination. Acta Neuropathol. 123, 247–258. 10.1007/s00401-011-0890-3 22009304 PMC3259380

[B47] Lee P. W. Yang Y. Racke M. K. Lovett-Racke A. E. (2013). Analysis of TGF-beta1 and TGF-beta3 as regulators of encephalitogenic Th17 cells: implications for multiple sclerosis. Brain Behav. Immun. 46, 44–49. 10.1016/j.bbi.2014.12.007 25499467 PMC4414699

[B48] Li L. Li D. P. Chen S. R. Chen J. Hu H. Pan H. L. (2012). Potentiation of high voltage-activated calcium channels by 4-aminopyridine depends on subunit composition. Mol. Pharmacol. 86, 760–772. 10.1124/mol.114.095505 25267719 PMC4244593

[B49] Liedtke W. Edelmann W. Chiu F. C. Kucherlapati R. Raine C. S. (1998). Experimental autoimmune encephalomyelitis in mice lacking glial fibrillary acidic protein is characterized by a more severe clinical course and an infiltrative central nervous system lesion. Am. J. Pathol. 152, 251–259. 9422542 PMC1858102

[B50] Linker R. A. Lee D. H. Flach A. C. Litke T. van den Brandt J. Reichardt H. M.. (2015). Thymocyte-derived BDNF influences T-cell maturation at the DN3/DN4 transition stage. Eur. J. Immunol. 45, 1326–1338. 10.1002/eji.201444985 25627579

[B51] Loeb J. A. Fischbach G. D. (1997). Neurotrophic factors increase neuregulin expression in embryonic ventral spinal cord neurons. J. Neurosci. 17, 1416–1424. 9006983 10.1523/JNEUROSCI.17-04-01416.1997 PMC6793741

[B52] Lucchinetti C. Bruck W. Parisi J. Scheithauer B. Rodriguez M. Lassmann H. (2000). Heterogeneity of multiple sclerosis lesions: implications for the pathogenesis of demyelination. Ann. Neurol. 47, 707–717. 10.1002/1531-8249(200006)47:6<707::AID-ANA3>3.0.CO;2-Q 10852536

[B53] Makar T. K. Trisler D. Sura K. T. Sultana S. Patel N. Bever C. T. (2008). Brain derived neurotrophic factor treatment reduces inflammation and apoptosis in experimental allergic encephalomyelitis. J. Neurol. Sci. 270, 70–76. 10.1016/j.jns.2008.02.011 18374360

[B54] McDermott K. W. Barry D. S. McMahon S. S. (2005). Role of radial glia in cytogenesis, patterning and boundary formation in the developing spinal cord. J. Anat. 207, 241–250. 10.1111/j.1469-7580.2005.00462.x 16185248 PMC1571535

[B55] Morsali D. Bechtold D. Lee W. Chauhdry S. Palchaudhuri U. Hassoon P.. (2013). Safinamide and flecainide protect axons and reduce microglial activation in models of multiple sclerosis. Brain 136, 1067–1082. 10.1093/brain/awt041 23518709

[B56] Ng K. Howells J. Pollard J. D. Burke D. (2013). Different mechanisms underlying changes in excitability of peripheral nerve sensory and motor axons in multiple sclerosis. Muscle Nerve 47, 53–60. 10.1002/mus.23455 23169153

[B57] Pekny M. Pekna M. (2004). Astrocyte intermediate filaments in CNS pathologies and regeneration. J. Pathol. 204, 428–437. 10.1002/path.1645 15495269

[B58] Ruck T. Bittner S. Simon O. J. Gobel K. Wiendl H. Schilling M.. (2014). Long-term effects of dalfampridine in patients with multiple sclerosis. J. Neurol. Sci. 337, 18–24. 10.1016/j.jns.2013.11.011 24290498

[B59] Rudy B. McBain C. J. (2001). Kv3 channels: voltage-gated K+ channels designed for high-frequency repetitive firing. Trends Neurosci. 24, 517–526. 10.1016/S0166-2236(00)01892-0 11506885

[B60] Rus H. Pardo C. A. Hu L. Darrah E. Cudrici C. Niculescu T.. (2005). The voltage-gated potassium channel Kv1.3 is highly expressed on inflammatory infiltrates in multiple sclerosis brain. Proc. Natl. Acad. Sci. U.S.A. 102, 11094–11099. 10.1073/pnas.0501770102 16043714 PMC1182417

[B61] Rush A. M. Dib-Hajj S. D. Waxman S. G. (2005). Electrophysiological properties of two axonal sodium channels, Nav1.2 and Nav1.6, expressed in mouse spinal sensory neurones. J. Physiol. 564, 803–815. 10.1113/jphysiol.2005.083089 15760941 PMC1464456

[B62] Sanchez J. A. Ho C. S. Vaughan D. M. Garcia M. C. Grange R. W. Joho R. H. (2000). Muscle and motor-skill dysfunction in a K+ channel-deficient mouse are not due to altered muscle excitability or fiber type but depend on the genetic background. Pflugers Arch. 440, 34–41. 10.1007/s004240000248 10863995

[B63] Schattling B. Steinbach K. Thies E. Kruse M. Menigoz A. Ufer F.. (2012). TRPM4 cation channel mediates axonal and neuronal degeneration in experimental autoimmune encephalomyelitis and multiple sclerosis. Nat. Med. 18, 1805–1811. 10.1038/nm.3015 23160238

[B64] Schnell S. A. Staines W. A. Wessendorf M. W. (1999). Reduction of lipofuscin-like autofluorescence in fluorescently labeled tissue. J. Histochem. Cytochem. 47, 719–730. 10.1177/002215549904700601 10330448

[B65] Segal J. L. Pathak M. S. Hernandez J. P. Himber P. L. Brunnemann S. R. Charter R. S. (1999). Safety and efficacy of 4-aminopyridine in humans with spinal cord injury: a long-term, controlled trial. Pharmacotherapy 19, 713–723. 10.1592/phco.19.9.713.31540 10391417

[B66] Sinha K. Karimi-Abdolrezaee S. Velumian A. A. Fehlings M. G. (2006). Functional changes in genetically dysmyelinated spinal cord axons of shiverer mice: role of juxtaparanodal Kv1 family K+ channels. J. Neurophysiol. 95, 1683–1695. 10.1152/jn.00899.2005 16319208

[B67] Smart S. L. Lopantsev V. Zhang C. L. Robbins C. A. Wang H. Chiu S. Y.. (1998). Deletion of the K(v)1.1 potassium channel causes epilepsy in mice. Neuron 20, 809–819. 10.1016/S0896-6273(00)81018-1 9581771

[B68] Stefoski D. Davis F. A. Fitzsimmons W. E. Luskin S. S. Rush J. Parkhurst G. W. (1991). 4-Aminopyridine in multiple sclerosis: prolonged administration. Neurology 41, 1344–1348. 10.1212/WNL.41.9.1344 1891078

[B69] Trapp B. D. Peterson J. Ransohoff R. M. Rudick R. Mork S. Bo L. (1998). Axonal transection in the lesions of multiple sclerosis. N. Engl. J. Med. 338, 278–285. 10.1056/NEJM199801293380502 9445407

[B70] Vacher H. Mohapatra D. P. Trimmer J. S. (2008). Localization and targeting of voltage-dependent ion channels in mammalian central neurons. Physiol. Rev. 88, 1407–1447. 10.1152/physrev.00002.2008 18923186 PMC2587220

[B71] Vergo S. Craner M. J. Etzensperger R. Attfield K. Friese M. A. Newcombe J.. (2011). Acid-sensing ion channel 1 is involved in both axonal injury and demyelination in multiple sclerosis and its animal model. Brain 134, 571–584. 10.1093/brain/awq337 21233144

[B72] Waxman S. G. (2006). Axonal conduction and injury in multiple sclerosis: the role of sodium channels. Nat. Rev. Neurosci. 7, 932–941. 10.1038/nrn2023 17115075

[B73] Waxman S. G. (2008). Mechanisms of disease: sodium channels and neuroprotection in multiple sclerosis-current status. Nat. Clin. Pract. Neurol. 4, 159–169. 10.1038/ncpneuro0735 18227822

[B74] Wu Z. Z. Li D. P. Chen S. R. Pan H. L. (2009). Aminopyridines potentiate synaptic and neuromuscular transmission by targeting the voltage-activated calcium channel beta subunit. J. Biol. Chem. 284, 36453–36461. 10.1074/jbc.M109.075523 19850918 PMC2794761

[B75] Xu M. Cao R. Xiao R. Zhu M. X. Gu C. (2007). The axon-dendrite targeting of Kv3 (Shaw) channels is determined by a targeting motif that associates with the T1 domain and ankyrin G. J. Neurosci. 27, 14158–14170. 10.1523/JNEUROSCI.3675-07.2007 18094255 PMC6673519

[B76] Ying Z. Roy R. R. Edgerton V. R. Gomez-Pinilla F. (2005). Exercise restores levels of neurotrophins and synaptic plasticity following spinal cord injury. Exp. Neurol. 193, 411–419. 10.1016/j.expneurol.2005.01.015 15869943

[B77] Zagha E. Lang E. J. Rudy B. (2008). Kv3.3 channels at the Purkinje cell soma are necessary for generation of the classical complex spike waveform. J. Neurosci. 28, 1291–1300. 10.1523/JNEUROSCI.4358-07.2008 18256249 PMC2657222

